# Architect or Destroyer: The Diversity of Glucocorticoid Receptor Signaling Pathways in the Brain

**DOI:** 10.3390/ijms27209027

**Published:** 2026-10-12

**Authors:** Liliia Shkarina, Raushana Khaybullina, Alexandra Primak, Kirill Bozov, Stalik Dzhauari, Maxim Karagyaur

**Affiliations:** Medical Research and Education Institute, Lomonosov Moscow State University, 27/10, Lomonosovsky Ave., 119192 Moscow, Russia; amon-ra1789@mail.ru (R.K.); primak.msu@mail.ru (A.P.); kir-bozov@yandex.ru (K.B.); stalik.djauari@yandex.ru (S.D.)

**Keywords:** glucocorticoid receptor, signaling pathways, brain tissue, epigenetics, stress, mental disorders

## Abstract

Glucocorticoids (GCs), acting through the glucocorticoid (GR) and mineralocorticoid (MR) receptors, are essential for brain development, stress adaptation, and metabolic homeostasis, yet their signaling exhibits context-dependent diversity yielding either beneficial or detrimental outcomes. This review synthesizes current knowledge on the multifaceted mechanisms through which GR integrates hormonal, metabolic, and stress signals to modulate transcriptional programs in the brain. We highlight three key levels of GR regulatory complexity: the diversity of DNA GR-binding motifs, GR-mediated epigenetic remodeling, and rapid non-genomic cascades. Clinical and experimental data reveal that while physiological GC levels are critical for normal maturation and function, excess or chronic exposure, especially during sensitive developmental windows, can disrupt neurogenesis, synaptogenesis, and myelination, predisposing individuals to cognitive, affective, and neurodegenerative disorders. We discuss promising therapeutic strategies based on selective modulation of MR/GR balance, interference with specific co-regulatory complexes, and targeted manipulation of DNMT/TET/HDAC cascades. Unlike most existing reviews—which address either (a) the MR–GR interaction as a whole receptor system, (b) non-genomic effects in isolation from genomic ones, or (c) the clinical manifestations of stress-associated disorders without an in-depth mechanistic analysis of GR targets—our manuscript attempts to integrate these three levels of GR regulation into a unified hierarchical system (binding motifs → epigenetic remodeling → the conditional nature of the boundary between genomic and non-genomic GR effects). We aim to explicitly demonstrate how these levels jointly determine cell-specific outcomes, rather than describing them in parallel as independent mechanisms. However, critical gaps remain in understanding the precise assembly of GR-co-regulator complexes in defined neuronal and glial populations, as well as the feasibility of selectively engaging beneficial GR signaling branches without systemic suppression of the hypothalamic–pituitary–adrenal axis. Addressing these questions is essential for developing next-generation therapies that harness GR’s architectural potential while avoiding its adverse or unwanted consequences.

## 1. Introduction

Living organisms are complex systems that are forced to keep their balance under the conditions of a changing external environment. One of the key systems of adaptation to changing conditions in humans and other mammals is the hypothalamic–pituitary–adrenal (HPA) axis, which plays an important role in mediating the link between stressful influences and epigenetic changes in the organism [1]. Epigenetic regulation, in its turn, acts as a conductor that determines the order in which particular genes are expressed, and this ensures the normal functioning and development of organs, tissues, and organisms as a whole. Dysregulation of gene expression may contribute to the emergence of diseases and, during ontogenesis, may lead to disturbances of development. In the brain, such changes lay the basis for the formation of mental and cognitive disorders.

One of the key effectors of the HPA axis are glucocorticoids (GCs) [2], which act through the glucocorticoid (GR) and mineralocorticoid (MR) receptors. Physiological levels of glucocorticoids are important for the everyday functioning of the organism: cortisol is the principal endogenous glucocorticoid of humans. In addition to taking part in adaptation to stress, it is one of the regulators of circadian rhythms, of metabolism, and of the immune response. During embryogenesis and at the early stages of ontogenesis, glucocorticoids play an important role in the normal maturation of brain structures [3,4], influencing such processes as proliferation, differentiation, migration, and cell survival. Experimental studies [5] and data from clinical observations demonstrate that exposure to glucocorticoids is able to induce epigenetic modifications and to change the expression of genes that are critically important for neurogenesis, synaptogenesis, and myelination, thereby determining the formation of the structure of the developing brain and the long-term activity of nerve cells.

The influence of GCs on organismal developmental programs is complex and multifaceted, as evidenced by a number of experimental and clinical studies [6,7,8], and the therapeutic use of synthetic GCs, which differ in their properties from natural ones, further complicates the overall picture. Thus, data obtained in in vitro models demonstrate that GCs can exert opposing effects on neurogenesis: from the suppression of the proliferation of neural progenitor cells to the stimulation of their division [6,8]. Enhanced proliferation has also been shown in vivo: a single administration of dexamethasone to pregnant mice increased the proliferation of individual populations of cortical neural progenitor cells, which led to a change in the number of neurons at birth and to anxiety-like and depression-like behavior in adult animals [9]. The direction of the effect may depend on the concentration of the hormone and on the receptor type: in human hippocampal progenitor cells, low concentrations of cortisol acting through MR enhanced proliferation, whereas high concentrations acting through GR suppressed it [6]. In children exposed in utero to synthetic GCs, thinning of the cerebral cortex has been revealed [7]. These effects can lead to long-term changes in brain structure and trigger psychiatric and cognitive impairments [4,7,10].

Thinning of the cortex, predominantly of the frontal regions, has also been described in children exposed in utero to maternal depressive symptoms [11,12,13]. One of the probable mediators of this effect is considered to be the maternal HPA axis [14]; thus, in women with high anxiety during pregnancy the association between cortisol levels in maternal plasma and in amniotic fluid becomes stronger, which indicates a change in the barrier function of the placenta [15]. At the same time, within the normal physiological range, a higher level of maternal cortisol in the third trimester is associated, on the contrary, with a greater cortical thickness and better cognitive performance in children aged 6 to 9 years [16], which emphasizes the dependence of the effects of GCs on their concentration and on the timing of exposure. Thus, an elevated level of maternal cortisol in early pregnancy is associated with slower development of infants during the first year of life, and in late pregnancy with accelerated development [17]; a higher level of maternal cortisol in early (but not in late) pregnancy is also associated with an increase in the volume of the right amygdala and with affective problems in girls at about 7 years of age [18].

The choice of drug is of particular importance: the main barrier to maternal glucocorticoids in the placenta is the enzyme 11β-HSD2 [15], which inactivates cortisol, corticosterone, and prednisolone, but not dexamethasone [19]. At the same time, the antenatal use of corticosteroids in women at risk of preterm delivery reduces perinatal mortality and, probably, the risk of developmental delay in children [20], and the reduction in the risk of neurodevelopmental disorders is most evident in children born extremely preterm (before 28 weeks of gestation) [21]. However, if after such exposure the child was born late preterm (at 34 to 36 weeks) or at term, this exposure is associated with an increased risk of neurocognitive and psychiatric disorders [21,22,23]. Therefore, according to the FIGO (Federación Internacional de Ginecología y Obstetricia) recommendations, antenatal corticosteroids are indicated in anticipated preterm delivery at 24 to 34 weeks of gestation, whereas in anticipated delivery at 34 to 36 weeks their routine use is not recommended [24].

These nuances and contradictions only emphasize how important it is to maintain the balance of GCs and to take the context into account: it is possible that the use of GCs is justified under certain circumstances [19], yet there is a probability that an excess of GCs in the tissues of the embryo during critical periods of development, caused by maternal stress, by pathological conditions, or by the use of synthetic analogues, may disturb the fine regulation of the molecular programs of neurodevelopment and provoke the development of pathology.

The study of the effects of glucocorticoids depending on the context, of the molecular mechanisms of signal transduction, and of the genes under their control opens up possibilities for targeted correction and prevention of a wide range of diseases. In the present work we have attempted to summarize the main signaling pathways of the glucocorticoid receptor and also to pay attention to the way in which they modulate cellular processes.

## 2. Literature Search Methodology

We utilized the PubMed, ScienceDirect, and eLIBRARY.RU databases, as well as the Google search engine. Additionally, we manually reviewed the reference lists of the identified reviews and original articles. Key search queries included combinations of the following terms: “glucocorticoid receptor” AND (“genomic” OR “non-genomic” OR “epigenetic” OR “GRE” OR “nGRE”); “GR” AND (“brain” OR “hippocampus” OR “neurodevelopment”); “mineralocorticoid receptor” AND “glucocorticoid receptor” AND “balance”; “corticosterone” OR “cortisol” AND (“stress” OR “PTSD” OR “depression”) AND (“DNA methylation” OR “epigenetic”). Priority was given to: (1) original studies providing direct mechanistic data (e.g., ChIP-seq, ATAC-seq, reporter constructs, knockout/knockdown models) over descriptive or associative studies; (2) more recent publications when multiple studies addressed the same topic, provided this did not necessitate the exclusion of seminal or priority studies; and (3) data derived from CNS models or neuronal cell lines over data from peripheral tissues, though peripheral data were included with the caveat that their extrapolation to the brain is limited. The final reference list comprises 258 sources, consisting of original experimental studies (cell models, research on rodents, organoids, and human cohorts) and review articles, in a ratio of approximately 61% to 39%. The publication date range spans more than 50 years, from 1975 to 2026, with a median publication year of 2015 and a mean of 2013, reflecting both foundational studies on GR mechanisms of action and the latest research in the field.

## 3. Genomic Effects of GR

Glucocorticoid and mineralocorticoid receptors (GRs and MRs) are expressed ubiquitously in the human brain: GR and MR mRNA is detected in all brain regions examined [25]. At the same time, their expression levels differ between regions: the GR/MR ratio is highest in the pituitary gland and the cerebellum and lowest in the hippocampus [25], whereas MR expression is highest in the hippocampus [26] (see also the Allen Brain Atlas, [27]). Species differences must also be taken into account: in rodents, MR is concentrated predominantly in limbic structures [28], whereas in primates both receptors are expressed to a greater extent in the cerebral cortex as well [29]. The expression of GR and MR in the human brain also depends on sex and age: according to a study of surgical material from patients with epilepsy, in the frontal and temporal cortex it is higher in women than in men, and in the temporal cortex it increases during childhood and reaches adult levels by puberty [30]. It is assumed that the uneven distribution of the receptors across brain regions, the differences in their affinity for GCs, in their molecular partners, targets and signaling pathways, as well as the ability of GR and MR to interact and to influence each other’s signaling, account for the whole diversity of GC effects [31,32,33,34].

For GR and MR as receptors of GCs, two main signaling pathways are distinguished: through modulation of gene expression directly or through molecular partners (these are the so-called “slow” genomic effects), and through activation or modulation of the activity of other signaling cascades and metabolic pathways (the “fast” non-genomic effects).

An important aspect of glucocorticoid signaling is the differential activation of GR and MR depending on cortisol levels. Owing to its high affinity, MR is already occupied at basal hormone levels, whereas GR is activated at the circadian peak of glucocorticoid secretion and during stress [35] (Table 1). MR-mediated effects maintain homeostasis and provide for the initiation of the stress response, whereas GR-mediated effects provide for its termination and for the subsequent adaptive reactions [32]. According to the concept of the MR:GR balance, an adaptive response to stress requires these two receptor systems to be in equilibrium, and a disruption of this balance impairs both the initiation of the stress response and its control, which may lead to neuroendocrine dysregulation and increase vulnerability to stress-related disorders [28,32,34]. In this review, we focus mainly on the signaling pathways, molecular targets, and effects of the glucocorticoid receptor in the brain.

The glucocorticoid receptor belongs to the evolutionarily conserved nuclear receptor subfamily 3, which also includes the receptors for other steroid hormones (estrogens, progesterone, androgens, and mineralocorticoids). The gene encoding GR (nuclear receptor subfamily 3, group C, member 1, *NR3C1*) is located on chromosome 5 at locus 5q31.

After binding its ligand, GR translocates from the cytoplasm into the nucleus, where, as a monomer, a dimer, or a tetramer [37], it interacts with specific nucleotide sequences of DNA, the glucocorticoid response elements (GRE), or with negative GREs (nGRE), triggering chromatin remodeling and changes in gene activity, that is, the slow “genomic effects”, which arise within 15 to 30 min and last up to several days. Binding of GR to a GRE is considered to activate gene transcription, whereas binding to an nGRE, on the contrary, to suppress it [38,39]. However, recent data indicate that binding of GR to an nGRE does not always lead to repression of the gene [40,41]. Moreover, the profile of genes that change their activity under the action of GCs may differ radically depending on the cell type and the state of the cells [42].

In addition to the binding sites of activated GR described above (GRE and nGRE), glucocorticoid receptor binding sites (GRBs) are known that contain a half GRE, atypical motifs, or no obvious GRE repeat, as well as composite forms of GRE coupled with binding sites for other transcription factors (TFs), which allows GR to bind DNA in the immediate vicinity of other DNA-bound transcription factors, for example AP-1 and NF-κB [31,42], modulating their activity [43,44]. In addition, GR can be associated with DNA regions not directly but through protein-protein contacts with other transcription factors [31]. In particular, such an association of GR has been shown with Stat3 [45], AP-1 [42,46], and the p65 subunit of the transcription factor NF-κB [47] (Table 2). In recent years, however, this concept has been criticized: new data indicate that GR apparently binds DNA in one form or another in all cases, and the previously described protein–protein interactions may have involved cryptic (hidden) half GRE sites [31]. Thus, GR is able to bind directly to AP-1 response elements even in the absence of AP-1 itself [43], as well as to cryptic GR binding sites within NF-κB response elements [44]. In the hippocampus, binding to a GRE apparently remains the main mode of GR binding to DNA [31].

Of interest, a number of studies have demonstrated that GR binding sites are on average located closer to genes with increased expression (a median distance of the order of 10 kb from the transcription start site, TSS) than to genes with decreased expression (of the order of 100 kb from the TSS) [40]. At the same time, most GR binding sites are located more than 10 kb from the TSS, and their influence on promoters is mediated by spatial chromatin loops [40].

The direction and the strength of the effect of GCs on the expression of a particular gene are determined by a large number of factors. In addition to the presence of GRE or nGRE binding sites in the regulatory regions of the gene, that is, in the promoter and in distant enhancers [40,42], an important role is played by the cellular context [31,52,53], which determines the qualitative and quantitative composition of the molecular partners of GR that may be expressed differently in different regions and cells of one and the same organ or tissue; by the activity of other signaling pathways capable of modulating GR function; by the concentration of GCs [6] and the duration of their action; by the metabolic state of the cell, on which the activity of coregulators and of chromatin-modifying enzymes depends, since these use acetyl-CoA, NAD+, S-adenosylmethionine, and ATP as substrates and cofactors [54,55]; by post-translational modifications and conformational changes in GR and of its partners [56]; and also by the state of chromatin [57,58].

**Figure 1:**
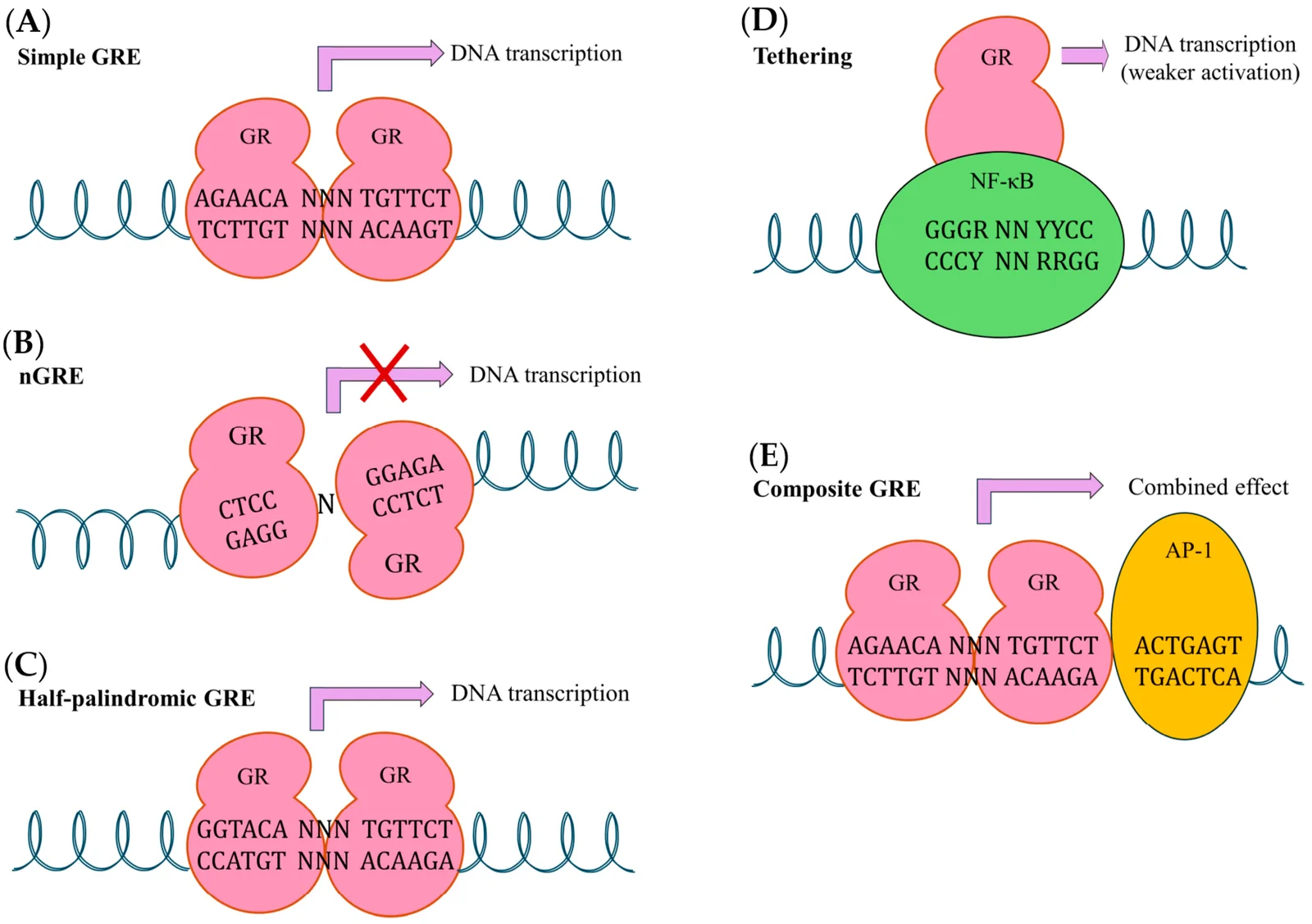
Architecture of glucocorticoid response elements and modes of GR action. The overall transcriptional outcome of GR action depends not only on the presence of glucocorticoids, but also on the architecture of the DNA binding site and the presence of partner transcription factors, which accounts for the tissue specificity and context dependence of glucocorticoid signaling. The type of binding site alone does not unambiguously determine the direction of the effect. (**A**) Classical GRE. Two GR monomers bind to a palindromic GRE sequence (AGAACA-NNN-TGTTCT) and form a homodimer that activates transcription of the target gene. This is the classical mechanism of direct positive regulation of gene expression by GR. (**B**) Negative GRE (nGRE). Two GR monomers bind to the inverted repeat CTCC(n)GGAGA, where n is 0–2 nucleotides (IR0–IR2). Dimerization is not possible, and binding occurs with negative cooperativity. The receptor recruits the corepressors NCoR/SMRT, which in turn recruit HDAC2 and HDAC3. This often results in repression (e.g., NR3C1, Tslp), but not always: binding to an nGRE does not necessarily repress the gene. (**C**) Non-consensus (degenerate) GRE. A GR dimer binds to a site that deviates from the consensus (example shown: GGTACA-NNN-TGTTCT). Such deviations may alter affinity and the efficiency of activation. This should not be confused with a GRE half-site, in which a GR monomer occupies a single AGAACA hexamer, including within κB and AP-1 motifs, and represses inflammatory genes. (**D**) Tethering. In the classical model, GR does not contact DNA directly but is held by protein–protein interactions with other transcription factors (e.g., NF-κB or AP-1) bound to their own sites. For some genes, direct binding of GR to cryptic half-sites within κB elements and AP-1 motifs has also been shown; therefore, the “pure” tethering model is currently being refined. (**E**) Composite GRE. Simultaneous binding of a GR homodimer to a classical GRE and of an adjacent transcription factor (e.g., AP-1) to its own response element (TGACTCA) produces a combined effect on transcription, integrating signals from different regulatory pathways.

The state of chromatin is a critically important parameter that influences the outcome of transcription [57,58,59]. GR forms complexes involving numerous proteins, including epigenetic modifiers of chromatin: the transcriptional complex of GR usually includes about ten coregulators, and in total dozens of coregulators are able to interact with GR, which gives rise to a great diversity of complexes and, accordingly, to differences in the outcomes of transcription [42,59]. The proteins that are part of the complexes with GR exert effects on transcriptional activity either on their own or through intermediaries, such as the p160 family of nuclear receptor coactivators (NCoA/SRC) and the nuclear receptor corepressors NCoR and SMRT, which are expressed in the brain, and the set of coregulators differs substantially between its regions [60] (Figure 1).

Steroid receptor coactivators, also known as SRCs and encoded by the genes *NCOA1* (SRC-1), *NCOA2* (SRC-2/TIF2/GRIP1), and *NCOA3* (SRC-3/AIB1), are able to bind chromatin-remodeling complexes (SWI/SNF), histone acetyltransferases (for example, p300/CBP, pCAF), readers of the epigenome, and the transcription initiation machinery, which promotes the activation of transcription of GR target genes [42,61,62] (Figure 1). In particular, knockout of SRC-1 has been shown to be associated with dysregulation of the GR target genes *Pomc* in the pituitary and *Crh* in the hypothalamus and the amygdala [59]. However, SRC-2, which is considered a coactivator of the glucocorticoid receptor, behaves in a dual manner when bound to GR: depending on the context, it can act both as an activator and as a repressor. Thus, GR together with SRC-2 acting as a corepressor interacts with DNA-bound AP-1 and NF-κB and suppresses the transcription of their target genes encoding pro-inflammatory cytokines [63]. This is set out in more detail in reviews [40,42,63,64]. Repression of transcription by the glucocorticoid receptor is accomplished through its interaction with corepressors such as NCoR1 and NCoR2 (SMRT), which recruit histone deacetylases (HDACs); NCoR complexes acquire their repressive activity mainly through the recruitment of HDAC3 [65].

GR is able to regulate gene transcription not only directly but also through the regulation of the transcription of genes encoding epigenetic modifiers. Thus, it has been shown that in the prefrontal cortex of rats subjected to repeated stress the association of GR with the GRE of the *Hdac2* promoter increased, which led to an increase in its expression. This was followed by an HDAC2-dependent decrease in the activity of the histone methyltransferase EHMT2 (G9a). That led to a loss of repressive histone methylation in the *Nedd4* promoter and to the activation of transcription of the E3 ligase NEDD4-1, which plays a critical role in the ubiquitination and degradation of ionotropic glutamatergic AMPA receptors. Inactivation of *Hdac2* restored the expression of AMPA receptors in animals subjected to repeated stress [66].

GR has the ability to influence DNA methylation and thereby to change gene expression. Thus, in rat pups subjected to the stress of maternal separation, an increase in plasma corticosterone was accompanied by increased expression of *Dnmt1* (which maintains methylation during DNA replication [67,68]), of *Dnmt3a* and of *Dnmt3b* (de novo methylation), as well as by increased activity of DNMT enzymes in the prefrontal cortex; at the same time, GR bound to the promoters of Dnmt3aand *Dnmt3b* and to a regulatory region in intron 3 of the *Dnmt3a* gene, and binding to the *Dnmt3b* promoter increased after stress [69]. In a primary culture of rat cortical neurons, corticosterone at high concentrations increased the expression of *Dnmt1*, *Dnmt3a*, and *Dnmt3b*, and the GR antagonist mifepristone abolished this effect [69]. Such changes may disturb the expression of downstream genes and affect the development and functioning of the brain. At the same time, under certain conditions glucocorticoids can cause a dose-dependent decrease in Dnmt1 expression, namely in AtT-20 pituitary cells under the action of dexamethasone and in the hippocampus of mice receiving corticosterone [70]; the loss of DNA methylation under the action of glucocorticoids is related to the function of DNMT1 during cell division [71].

Enzymes of the TET family (Ten-Eleven Translocation methylcytosine dioxygenases), which participate in active DNA demethylation, are able to interact directly with a number of nuclear receptors: in HEK293T cells it has been shown that TET3 binds the thyroid hormone receptors TRα1 and TRβ1, as well as the estrogen-related receptor ERRα and the androgen receptor AR [72]. Using TRα1 as an example, it has been established that TET3 reduces the ubiquitination and degradation of the receptor and stabilizes its association with chromatin, and this effect does not require the enzymatic activity of TET3, that is, it is not related to DNA demethylation [72]. An interaction with TET enzymes has been shown for GR as well, although outside the central nervous system (CNS): in human tolerogenic dendritic cells TET2 co-immunoprecipitates with GR, and the GR signal is detected in the vicinity of CpGs that are specifically demethylated under the action of glucocorticoids [73]; in HEK293T cells an interaction of GR with proteins of the TET family, primarily with TET2, has been revealed [74]. It had been shown earlier that activation of GR causes local active DNA demethylation in the enhancer of a target gene, which persists after hormone withdrawal and enhances the response to its repeated action [75,76]. Whether similar interactions occur with the participation of MR, and also in brain cells, is not yet known [77]. At the same time, in progenitor cells of the human fetal hippocampus dexamethasone increased the expression of TET1 and UHRF1, enzymes associated, respectively, with demethylation and with remethylation of DNA, which is consistent with changes in DNA methylation in both directions [78]. However, after a 20-day washout period the changes in TET1 and UHRF1 expression disappeared, whereas the changes in methylation persisted; according to the authors, the persistent changes in methylation are due not to a sustained change in the expression of these enzymes but, possibly, to their locus-specific recruitment [78]. Thus, the ability of TET enzymes to interact with nuclear receptors, including GR, the ability of GR to cause local DNA demethylation, and the persistence of methylation changes after the normalization of enzyme expression allow one to suppose that the recruitment of TET to the regulatory regions of genes with the participation of GR may contribute to the formation of long-term epigenetic changes in the brain associated with mental disorders; however, this hypothesis requires experimental verification.

Such complex and multilevel regulation of the epigenome by the glucocorticoid system, as well as its dependence on a large number of factors, may lead to changes in DNA methylation in opposite directions. In a number of studies demethylation predominated: thus, in young male mice receiving corticosterone, about 70% of the sites that changed their methylation, both in the blood and in the hippocampus, lost methylation [79]. However, in human hippocampal progenitor cells the persistent changes in methylation after exposure to dexamethasone occurred in both directions: a predominance of demethylation was noted only in GREs, whereas in enhancers, in bivalent regions, and in regions flanking the transcription start site, hypermethylation and demethylation occurred with approximately equal frequency [78]. The authors relate the opposite directions of these changes to the simultaneous increase in the expression of TET1 and UHRF1 (see above). The decrease in methylation apparently occurs not only passively during cell division but also actively [75], through the involvement of the methylcytosine dioxygenases TET1 to TET3 [68,80,81,82,83].

Similarly, dexamethasone caused genome-wide DNA hypomethylation in neural stem cells isolated from the cortex of rat embryos, which was accompanied by a decrease in Dnmt3a expression and an increase in Tet3 expression; these changes persisted in daughter cells that had never been directly exposed to dexamethasone [83]. A similar picture, that is, global DNA hypomethylation, a decrease in *Dnmt3a* expression, and an increase in the expression of *Tet1* to *Tet3*, was also observed in vivo, in the cerebral cortex of 3-day-old mouse pups after intrauterine exposure to dexamethasone [83]. Knockdown of GR abolished the dexamethasone-induced decrease in *Dnmt3a* expression but not the increase in Tet3, that is, the activation of *Tet3* apparently does not depend on GR. In the opinion of the authors, a GR-mediated action can explain the direct effect of dexamethasone on Dnmt3a but not the heritable changes in the daughter cells; the key role in maintaining them is assigned by the authors to *Tet3*, knockdown of which in the parental cells abolished the dexamethasone-induced decrease in *Dnmt3a* expression and increase in the expression of *Dkk1*, a factor required for the differentiation of neurons [83]. The authors believe that this mechanism may participate in the epigenetic programming caused by early-life events that are accompanied by high levels of GCs, which may contribute to long-term disturbances of the development of the nervous system.

In addition to interacting with DNA directly, GR is able to modulate the activity of dozens of different tissue-specific regulators (simultaneously or at different times, associating with them on DNA or through protein–protein interactions), which makes it possible to generate a broad spectrum of transcriptional complexes, each of which can trigger a unique transcriptional response [42]. In particular, GR is able to bind the dimer of the transcription factor AP-1 (c-Fos/c-Jun) through an interaction with c-Fos, which leads to the suppression of its binding to DNA and of its transcriptional activity; another composition of AP-1, the c-Jun/c-Jun homodimer, is suppressed by GR considerably more weakly [63]. Moreover, in a model cell system it has been shown that with c-Jun/c-Jun homodimers GR can act synergistically, activating AP-1-dependent promoters that contain no GR binding sites [46], so that the direction of the effect of GR on AP-1-dependent genes is determined by the composition of the AP-1 dimer. In addition, DNA-bound AP-1 maintains chromatin accessibility and thereby facilitates the binding of GR to regulatory regions of the genome [84]. Binding of GR almost always occurs in chromatin regions that were accessible even before the action of the hormone, yet the binding itself locally increases this accessibility [49]. At the same time, GR causes nucleosome shifts in enhancers of all types and recruits the remodeling complex BRG1 (the ATPase component of the SWI/SNF chromatin remodeling complex), and in nucleosomal enhancers it behaves like a pioneer factor, binding nucleosomal DNA and recruiting BRG1 for chromatin remodeling [57].

Langlais et al. demonstrated suppression of the transcription that STAT3 usually induces in the case where GR joins STAT3 that is already bound to DNA. In the remaining cases, the joint action of GR and STAT3 led to a more pronounced activation of transcription than the action of each factor separately. Thus, the binding of GR and STAT3 to closely spaced regulatory elements (within about 200 bp) stabilized their interaction and enhanced the recruitment of coactivators (for example, p300), which increased the efficiency of transcription; the formation of a composite regulatory element containing overlapping binding motifs for STAT3 (SBE) and GR (GRE) separated by 8 base pairs showed a particularly strong response to dexamethasone, that is, it provided synergistic activation. In the opposite case, when Stat3 joined GR that was already bound to DNA, a synergistic effect was also observed [45].

Taken together, these data allow one to suppose that the factors regulating the plasticity of the GC response networks are a kind of allosteric effector: each of them contributes to the formation of a specific conformation of the receptor that reflects a certain integrated context. As a result, conformational changes in GR create unique surface patterns recognized by the environment of GR, and this underlies the adaptive rearrangement of GR-dependent transcriptional programs [42]. It is of interest that GR can interact not only with accessible DNA but can also behave as a pioneer factor, triggering chromatin remodeling in nucleosome-enriched enhancers through the recruitment of the ATPase BRG1, which is part of the SWI/SNF complex [57].

When planning and analyzing the results of an experiment, one cannot fail to take into account the temporal parameters of GC exposure, since the transcriptional response is kinetically diverse: under constant action of the hormone, transcription may change over time, up to an alternation of phases of activation and repression [40,64]. The reason may lie in a change in interaction partners; in the time during which a TF is able to bind DNA; in a change in chromatin accessibility; in the presence of feedback loops; and also in the fact that GR induces the expression of its own cofactors, which then either enhance or, on the contrary, suppress its action [40]. Thus, in rat hippocampal slices and in neuronally differentiated PC12 cells, only repressed genes were detected 1 h after GR activation, whereas after 3 h predominantly, or at least in part, activated genes were detected; after 5 h the response had almost returned to the initial level [85,86]. Presumably, the earliest changes in gene expression, detected during the first hour after GR activation, are realized through transrepression, which precedes a later wave of transactivation [86]. An increase in expression occurs later, since it requires the recruitment of cofactors and chromatin remodeling [85]. However, rapid activation of transcription by the glucocorticoid receptor is also possible, both through its direct interaction with components of the general transcriptional machinery and through binding to glucocorticoid response elements; nevertheless, at early times after GR activation the suppression of gene expression still predominates [85,87]. At the same time, in human lung adenocarcinoma cells the repression of genes, on the contrary, developed later than their activation [88], which indicates that the temporal profile of the response depends on the cell type. It should also be taken into account that the later transcription of some genes may be due not to GR itself but to proteins whose expression it had induced; thus, 1 h after GR activation most changes in expression were not blocked by an inhibitor of protein synthesis and were primary, whereas after 3 h a mixture of primary and secondary responses was already detected [40,85]. Therefore, in order to increase the scientific reliability of an experiment, it is necessary to distinguish between primary and secondary transcriptional responses to activation of the glucocorticoid receptor [85].

## 4. Non-Genomic Effects of GR

In addition to the “slow” genomic effects realized by GR as a transcription factor, it is able to initiate “fast” effects that do not involve binding to DNA either directly or through interaction with other TFs [89]. Non-genomic effects may be mediated both by cytosolic GR and by membrane-bound GR (mGR), and at high concentrations of glucocorticoids also by mechanisms that do not depend on GR: through the non-specific insertion of glucocorticoid molecules into cell membranes, which affects cation transport and proton leakage in mitochondria, and through an increase in the intracellular level of second messengers, namely inositol trisphosphate, cAMP, and calcium ions [89,90,91]. The existence of mGR has been shown, in particular, on human monocytes and B lymphocytes [92], as well as on a number of cell lines, including leukemia cells, where its activation caused rapid changes in the proteome [93]. At the same time, the molecular nature of mGR remains a subject of discussion [94]: according to some data it is an isoform of GRα, according to others an isoform of GRγ [90]. In addition, a possible role of G protein-coupled receptors (GPCRs) as mediators of some of the rapid effects of glucocorticoids is discussed [95].

The non-genomic activity of GR initiates diverse molecular signaling pathways, for example endocannabinoid signaling, ERK1/2, the glucocorticoid-induced non-genomic pathway involving mGR and nitric oxide (NO) [96,97], and pathways leading to modulation of the intracellular level of Ca2+ [89,98]. In addition, besides the possibility of direct activation of downstream intracellular signaling pathways, a number of studies point to cooperation between mGRs and GPCRs [89,95]. All this leads to rather diverse effects depending on the cellular context, the dose, the duration and the number of administrations, and also on environmental conditions.

Non-membrane GR, being in the cytosol, is able to realize its activity through protein–protein interactions in the cytoplasm or through translocation into mitochondria [91,99]. Thus, after ligand binding, cytosolic GR interacts directly with the signaling kinases SRC and PI3K/Akt and with the mitogen-activated protein kinases MAPK, which within a few minutes suppresses the activity of NF-κB and AP-1 without the participation of transcription [100]. An understanding of the mechanisms of the cytoplasmic events associated with GR is important for the creation of methods and means of therapeutic intervention in inflammation and in stress-related disorders. Such mechanisms include the regulation of NF-κB activity by the glucocorticoid receptor, which involves both direct and indirect interactions between NF-κB and GR [100,101,102]. Thus, GR increases the affinity of IκB for the NF-κB complex; in addition, even in the absence of corticosteroid signaling, cytosolic GR is able to interact with the NF-κB subunits p65 and p50, as well as with IκB, and to suppress the nuclear translocation of NF-κB [102]. Activated GR can also accelerate the export of the p65 subunit from the nucleus, decreasing its transcriptional activity [100]. It has also been shown that NF-κB subunits and proteins of the cascade of its activation are able to interact with chaperones that regulate the nuclear translocation of GR; in particular, in hippocampal neurons NF-κB binds the chaperone Hsc70; however, the functional significance of these interactions in vivo remains largely undetermined [102].

A curious signaling mechanism triggered upon activation of GR is the release of GR partner proteins, which can also act as regulators of metabolic processes and as inducers of signaling cascades (for example, the release of arachidonic acid, a structural element of cell membranes and a precursor of lipid mediators that regulate inflammatory reactions and functions, including those of the nervous system). It is known that ligand-unbound GR is retained in the cytoplasm as part of a multiprotein complex that includes heat shock proteins (HSP), co-chaperones (p23, p60), immunophilins (FKBP), and a number of kinases, including the non-receptor tyrosine kinase SRC. In A549 lung adenocarcinoma cells, dexamethasone caused the release of both GR and Src from the complex with HSP90 within as little as 5 min, and these were independent events: geldanamycin, which prevents the nuclear translocation of GR, blocked the release of GR but not of SRC [103]. The rapid suppression of the release of arachidonic acid induced by epidermal growth factor (EGF) did not require the nuclear translocation of GR or its genomic effects, but was abolished by an inhibitor of kinases of the SRC family; the authors concluded that this role is performed precisely by the release of SRC, which triggers the phosphorylation of lipocortin 1 (annexin A1) [103].

Non-genomic effects of glucocorticoids may also indirectly influence nuclear processes. Thus, it has been shown that psychological stress activates NMDA receptors (NMDAR) in hippocampal neurons and triggers the NMDAR-ERK-MAPK pathway, which leads to the translocation of activated pERK1/2 into the nucleus, where GR can bind it even before GR begins to interact with DNA as a transcription factor. This enhances the ability of pERK1/2 to activate the nuclear kinases MSK1/2 and ELK-1, which leads to the phosphorylation and acetylation of the amino acid residues Ser10 and Lys14 of the histone H3 protein (H3S10p-K14ac). Acetylation is apparently accomplished through the recruitment of the histone acetyltransferase p300 by phosphorylated ELK-1 (pElk-1). This leads to chromatin remodeling in a number of genes, including immediate early genes such as FOS (the c-fos protein) and EGR1 (early growth response protein 1) [104,105]. It is assumed that the subsequent transcription of these genes contributes to the consolidation of the memory associated with the experienced event. This scenario is classified as a non-genomic (pre-genomic) effect of GR, since it is realized within tens of minutes after stress but depends on protein–protein interactions of GR with components of the signaling cascade.

It is important to note that the rapid non-genomic effects of GR are limited in time. Termination of the signaling is apparently provided by several mechanisms. One probable mechanism is the glucocorticoid-inducible dual specificity phosphatase 1 (DUSP1), also known as mitogen-activated protein kinase phosphatase 1 (MKP-1), a key element of negative feedback that interrupts further signal transduction: it dephosphorylates and thereby inactivates ERK1/2 and other MAP kinases (shown mainly for immune cells) [106]. Remarkably, in this case the rapid effects are limited by a genomic mechanism, namely by the induction of transcription of the *DUSP1* gene. In parallel, the membrane receptor (mGR) itself, like other G protein-coupled receptors, may undergo desensitization and internalization; however, the specific molecular mechanisms of these processes in brain neurons remain insufficiently studied. It may be supposed that a disturbance of these mechanisms of signal termination under conditions of chronic stress leads to a pathological persistence of non-genomic cascades. The significance of the latter is supported by the fact that in rodents blockade of the links of the GR→ERK→MSK→H3S10p-K14ac pathway disrupts the consolidation of the behavioral response to stress; a role of this pathway in the pathogenesis of post-traumatic stress disorder (PTSD) is assumed [105]. Since the release of arachidonic acid serves for the synthesis of inflammatory mediators [103], a disturbance of its rapid GR-mediated suppression may potentially contribute to an enhancement of inflammatory reactions. Indirect confirmation of the significance of this mechanism is provided by data obtained upon knockout of the annexin A1 gene, the effector through which the rapid suppression of arachidonic acid release is realized. *Anxa1*−/− cells overexpress cPLA2, sPLA2, and COX-2 and release more arachidonic acid and prostaglandin E2, and dexamethasone no longer suppresses this release [107], while in *Anxa1*−/− mice the inflammatory response is more severe and partially or completely loses its sensitivity to glucocorticoids [108]. It should be taken into account that these data were obtained in models of peripheral inflammation.

## 5. Targets of GR Action in the CNS

The combination of the methods ChIP-seq (chromatin immunoprecipitation sequencing), RNA-seq (RNA sequencing), scRNA-seq (single-cell RNA sequencing), and ATAC-seq (assay for transposase-accessible chromatin sequencing) has made it possible to move from a list of GR binding sites to its functional targets, that is, to the genes in which GR not only occupies regulatory regions but also changes the level of transcription. Thus, genome-wide mapping of GR binding in PC12-derived neuronal cells revealed 1183 binding sites, of which 58% contained a GRE, while the remaining 42% did not contain one and apparently bind GR indirectly, through other transcription factors; a change in the expression of the nearest genes after GR activation could be confirmed in approximately half of the cases examined [109]. Single-cell sequencing has shown that the response to GR activation is heterogeneous and depends on the cell type: in cerebral organoids modeling the developing brain, GR was expressed in all cell types, whereas the most pronounced changes in the transcriptome affected neurons and progenitor cells [53]. The integration of scRNA-seq and scATAC-seq data made it possible to reconstruct the regulatory networks through which chronic exposure to glucocorticoids exerts a long-lasting influence on the specialization of cells, in particular with the participation of the transcription factor PBX3 [110].

Below we consider some of the most frequently mentioned genes involved in the realization and the regulation of the glucocorticoid response, and also the way in which stressful experience and the epigenetic modifications associated with it change the activity of these genes, converting short-term molecular events into long-term rearrangements of neuronal networks and into an increased vulnerability to neuropsychiatric disorders.

### 5.1. Genes of GR Self-Regulation

*FKBP5* is the gene of the co-chaperone protein FKBP51 (FK506-binding protein 51). FKBP51 is part of an ultrashort negative feedback loop that regulates the activity of the glucocorticoid receptor: it retains GR in the cytoplasm, preventing its translocation into the nucleus. GR induces the transcription of *FKBP5* through interaction with steroid hormone response elements. The functional GREs of the *FKBP5* gene are distributed over a region more than 100 kb long, from the area upstream of the promoter to introns 2, 5, and 7; these enhancer elements activate transcription through the formation of three-dimensional chromatin loops [111,112].

Under the action of an increased concentration of GC, GR-mediated demethylation of *FKBP5* and its abnormal overexpression occur, apparently through a decrease in the expression of Dnmt1 [70,71]. Demethylation of *FKBP5* presumably disturbs the interaction of the corresponding demethylated DNA with the MeCP2 protein, which realizes its repressor function by recruiting a histone deacetylase. This leads to persistent expression of *FKBP5*. The increased activity of FKBP51 excessively limits the nuclear translocation of GR and entails a decrease in GR-dependent transcriptional activity, a disturbance of the feedback of the HPA axis, and an insensitivity to GCs, which contributes to the emergence of stress-related disorders [71,112,113]. A loss of methylation of the *FKBP5* gene under the influence of glucocorticoids has been observed in the hippocampus (most pronouncedly in the dentate gyrus), in the hypothalamus, the amygdala, the prefrontal cortex, and the blood, as well as in the HT-22 neuronal cell line and in AtT-20 pituitary cells [70,71,114]. At the same time, in brain regions demethylation affects the CpGs of the fifth intron, whereas in blood a loss of methylation in the first intron has been described [71].

**Figure 2:**
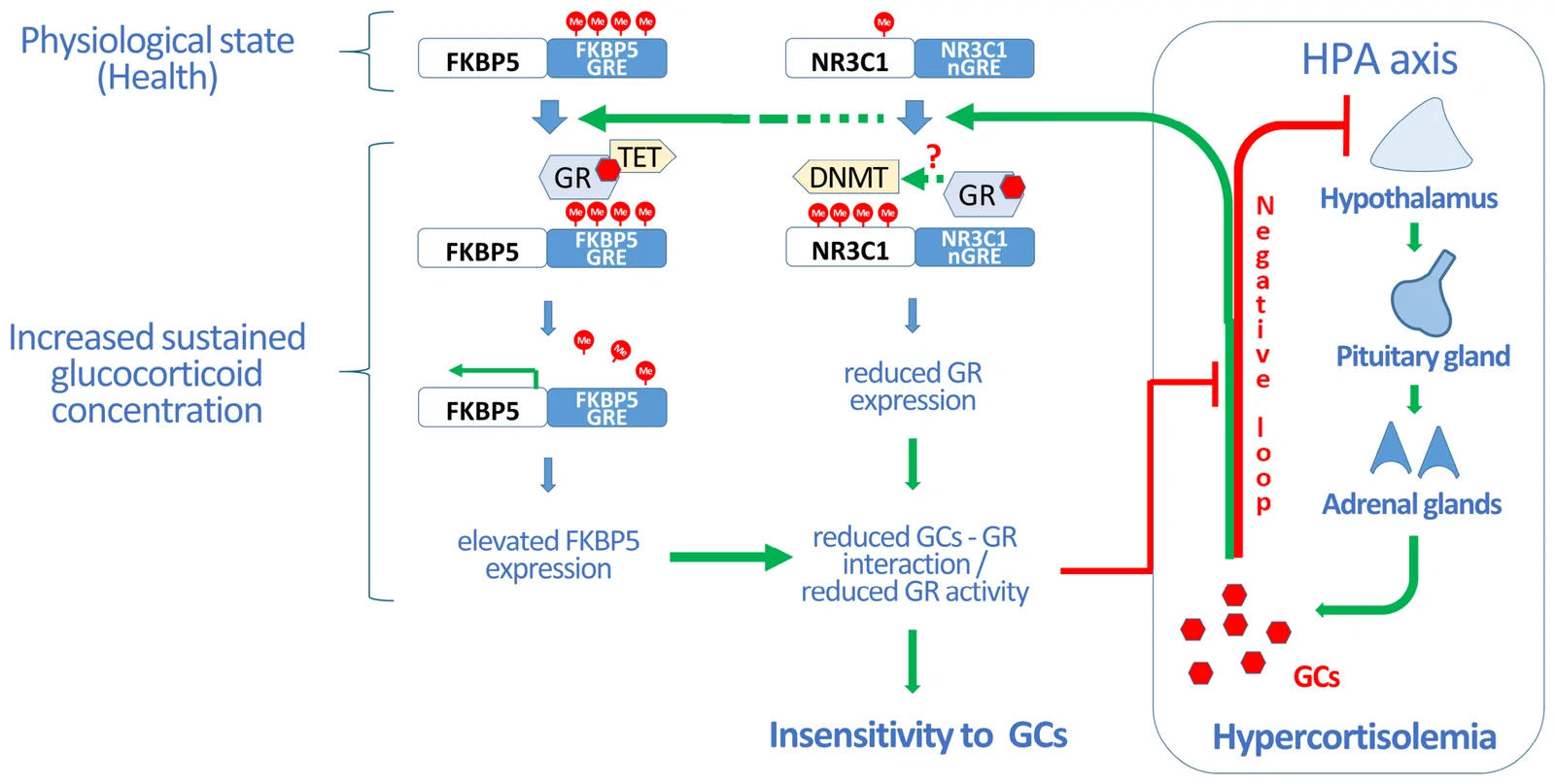
Molecular mechanisms of GR-dependent self-regulation in physiological state and during its impairment. FKBP5 encodes the co-chaperone FKBP51, which retains GR in the cytoplasm and thus forms an ultrashort negative feedback loop. At elevated GC levels, GR-mediated demethylation of FKBP5 regulatory CpGs occurs, sustaining gene expression. Excess FKBP51 limits nuclear translocation of GR, reduces GR-dependent transcription, and leads to GC insensitivity and impaired HPA axis feedback. Ligand-bound GR negatively regulates its own gene through an nGRE in exon 6. Under chronic or early-life stress, hypermethylation of NR3C1 regulatory regions (involving DNMT3a) may lead to a further reduction in GR expression, impaired HPA axis feedback, and insensitivity to GCs. The involvement of GR in recruiting DNMT to the NR3C1 nGRE is hypothetical and has not been directly demonstrated.

The expression of the glucocorticoid receptor gene itself (*NR3C1*) is regulated by glucocorticoids on the principle of negative feedback: ligand-bound GR is recruited to exons 6 and 8 of its own gene, and a functional nGRE is located in exon 6, so that, owing to long-range chromatin interactions, a repressor complex of GR, NCoR1, and HDAC3 assembles at the transcription start site and suppresses the initiation of transcription [115]. Early life stress is associated with an increase in the methylation of the promoter regions of *NR3C1* and with a decrease in its expression, which is regarded as one of the causes of hyperactivation of the HPA axis [116]. In the dentate gyrus of the rat hippocampus, the level of GR mRNA decreases after acute stress caused by forced swimming. The authors explain this decrease by a combination of two mechanisms: an epigenetic one, in that after stress the binding of DNMT3a to a region of exon 1_7_ of the *NR3C1* gene increases and the methylation of individual CpGs in this region rises; and a post-transcriptional one, in that the expression of miR-124a, which lowers the level of GR mRNA, increases. At the same time, the binding of GR to the nGRE in exon 6 does not change after stress [117] (Figure 2).

### 5.2. Genes Regulating the HPA Axis

The transcription of the genes *CRH* (corticotropin-releasing hormone) and *POMC* (pro-opiomelanocortin), the precursor of adrenocorticotropic hormone (ACTH) in the pituitary, is regulated by GR on the principle of feedback within the hypothalamic–pituitary–adrenal axis. Their expression is suppressed in order to reduce the production of GCs after stress.

In the promoter of the *CRH* gene, a negative glucocorticoid response element (nGRE) has been identified that contains adjacent binding sites for GR and AP-1 (c-Jun/c-Fos). GR and AP-1 interact directly with these nucleotide sequences, providing the feedback of the hypothalamic–pituitary–adrenal axis [51].

**Figure 3:**
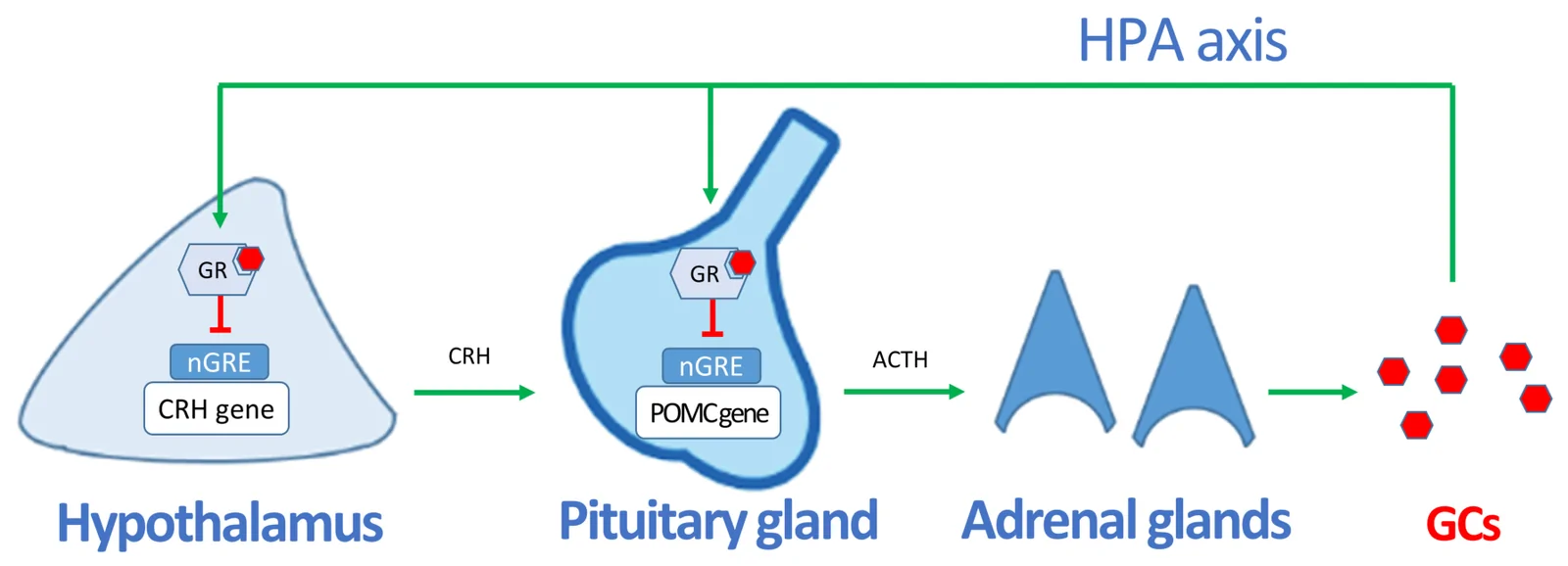
Molecular mechanisms of GR-dependent self-regulation in physiological state and during its impairment. Ligand-bound GR represses *CRH* and *POMC* transcription via negative feedback; an nGRE with adjacent GR and AP-1 sites has been described for *CRH*, whereas *POMC* may be repressed through an nGRE and/or a NurRE-dependent pathway. At normal GC levels this maintains the balance of the CRH → POMC/ACTH → GCs axis. When GR function is reduced (e.g., by FKBP51 or decreased *NR3C1* expression), repression of *CRH* and *POMC* is weakened, feedback is impaired, and GC secretion remains elevated.

In the promoter of the *POMC* gene an nGRE has also been identified: in the rat it is located in the region of −63 bp and provides the binding of three GRs, a homodimer with the subsequent attachment of a monomer. In addition to the direct action through the nGRE, GR also suppresses the transcription of *POMC* without direct binding to DNA: it interacts through protein-protein contacts with factors of the Nur family bound to the NurRE element (Figure 3). This process requires the participation of BRG1 and is accompanied by the recruitment of the histone deacetylase HDAC2, which leads to a decrease in histone acetylation at the promoter and in the body of the gene, with subsequent repression of transcription. The loss of nuclear expression of *BRG1* or *HDAC2* may explain glucocorticoid resistance in more than 50% of adenomas in Cushing’s disease, both in dogs and in humans. Which of the two mechanisms predominates in vivo is not yet clear: mapping of glucocorticoid-sensitive regions in AtT-20 cells pointed to NurRE rather than to the nGRE [50].

### 5.3. Genes of Brain Morphogenesis

Serum/glucocorticoid regulated kinase 1 (SGK1) is a serine/threonine kinase that participates in the regulation of ion transport, neuronal excitability, synaptic plasticity, and the proliferation and differentiation of cells. The *SGK1* gene contains a GRE sequence, and this is what provides for the regulation of SGK1 expression by the glucocorticoid receptor [118]. In a model based on a line of human hippocampal progenitor cells, SGK1 was identified as an important mediator capable of reducing the proliferation of progenitor cells and of suppressing neurogenesis in the hippocampus, in particular through inhibition of the Hedgehog signaling pathway [119].

A decrease in *SGK1* expression has been found in the postmortem prefrontal cortex of people with PTSD, in a small sample (six cases and six controls), by microarray analysis with confirmation by quantitative PCR; the authors note that this result requires verification in a larger number of cases [120]. A similar decrease in SGK1 in the prefrontal cortex is reproduced in rats in the learned helplessness model, and suppression of SGK1 activity in the medial prefrontal cortex by itself induces behavior characteristic of traumatic stress, reduces the density of dendritic spines, and disturbs synaptic transmission [120]. An increase in *SGK1* expression, on the contrary, is associated with major depressive disorder (MDD) [121]: an increase in the level of *SGK1* RNA was noted in patients with depression in the blood and in the hippocampus, and also in the hippocampus after modeling of prenatal stress [119,122]. At the same time, no changes in *SGK1* expression in the prefrontal cortex have been described in MDD, which may indicate that this feature is specific to PTSD [120].

The product of the *BDNF* gene (brain-derived neurotrophic factor) is a protein belonging to the neurotrophin family; it plays a key role in the development, functioning, and survival of the neurons of the central and peripheral nervous system. Chronic exposure to glucocorticoids leads to suppression of *BDNF* expression in the hippocampus, which is one of the links in the pathogenesis of depressive states. The action of corticosteroids on BDNF is mediated by both of their receptors, and in opposite directions: in a culture of rat cortical neurons aldosterone increased, whereas dexamethasone decreased, the level of *Bdnf* mRNA and protein, while endogenous corticosterone, which binds both receptors, produced a biphasic effect [123]. The contribution of GR itself has been shown directly: in neuron-like hippocampal cells the GR antagonist RU486 completely abolished the dexamethasone-induced decrease in the level of BDNF transcripts [124].

However, the action of GR on BDNF is not limited to the regulation of the transcription of its gene. The effects of glucocorticoids can be traced along the whole path from the gene to the signal in the postsynaptic neuron: at the levels of transcription, of translation, of the post-translational processing of proBDNF into mature BDNF, of the sorting and transport of the neurotrophin into dendrites or axons, and, finally, of signal transduction after the binding of BDNF to the TrkB and p75NTR receptors [125]. The direct participation of GR has been established at three of these levels. At the level of transcription, GR acts on the promoters of the *BDNF* gene, either through putative GREs or through other transcription factors, primarily AP-1 and CREB. At the level of processing, GR induces the transcription of the gene of plasminogen activator inhibitor 1 (PAI-1) and thereby shifts the balance of the tissue plasminogen activator (tPA) system, on which the proportion of mature BDNF depends. At the level of signal transduction, GR physically interacts with the TRKB receptor and supports the activation of the PLCγ pathway: chronic action of dexamethasone reduces the amount of GR itself while the level of MR remains unchanged, weakens the interaction between TRKB and GR, and decreases the BDNF-dependent release of glutamate [126]. The influence turns out to be mutual: not only does GR regulate BDNF, but BDNF also tunes the receptor itself. BDNF, acting through TRKB, causes phosphorylation of GR at the residues Ser155 and Ser287, which increases the occupancy of GR at the promoters of target genes and the recruitment of cofactors to them. As a result, the joint action of BDNF and dexamethasone activates a particular set of GR-dependent genes associated with the growth and differentiation of neurons and enhances the induction of many dexamethasone-sensitive genes; the level of GR phosphorylation in vivo depends on the amount of BDNF and TRKB and on stress [127].

For a number of other effects, the participation of GR is probable but has not been shown directly. Thus, dexamethasone disturbs the binding of TKBB to the adaptor protein SHP2 and weakens the MAPK/ERK pathway, while the level and the phosphorylation of SHP2 itself do not change, an inhibitor of SHP2 reproduces the action of dexamethasone, and overexpression of SHP2 abolishes it; the participation of the glucocorticoid receptor was not examined in that work [128]. Dexamethasone also reduces the BDNF-induced increase in the level of microRNA-132 and, following it, the level of the postsynaptic glutamate receptors NR2A, NR2B, and GLUR1 [129]. Changes in DNA methylation and of histone modifications in the promoters of *Bdnf*, primarily in the promoter of exon IV, have been described after early life stress accompanied by an increase in the level of glucocorticoids; however, the contribution of GR to the placement of these marks was not established, and the direction of the changes depends on age, on sex, and on the brain region [130]. Below we consider in more detail the two best studied levels, the transcriptional one and the post-translational one.

At the level of transcription, the action of GR through other transcription factors is the best characterized. Thus, in db/db mice with a chronically elevated level of corticosterone, GR binds the AP-1 complex and suppresses its action at promoters I and IV of the *Bdnf* gene [131]. In neuron-like mouse hippocampal cells, GR is recruited to a short (275 bp) region of the promoter of exon IV that contains no classical GRE [124], where it interacts with the response element of the EGR1 protein in the area of the transcription start site of the *Bdnf* gene and at the same time physically binds EGGR itself [132]; mutations of the AP-1 sites in this region did not abolish the suppression by dexamethasone, that is, the contribution of one or another partner apparently depends on the model and on the region of the promoter [124].

In addition, GR regulates the post-translational maturation of BDNF through tPA and its main inhibitor PAI-1 [133]. In the PAI-1 gene (*SERPINE1*), a functional GRE has been identified that mediates the induction of its transcription by glucocorticoids [134], and acute stress increases the expression of PAI-1 in the hippocampus and thereby modulates the activity of tPA [135]. tPA, through the activation of plasmin, provides for the cleavage of proBDNF into mature BDNF, which is necessary for the late phase of long-term potentiation (LTP) in the hippocampus [136]; in transgenic mice with increased expression of tPA in neurons, LTP is enhanced and prolonged, and spatial learning is improved [137]. Under moderate stress, GCs increase the expression of tPA in the hippocampus, which through the TrkRKreceptor and the ERK1/2 MAPK cascade enhances the formation of memory, whereas under very intense stress high levels of GCs increase the production of PAI-1, which blocks tPA; in mice this led to the formation of a PTSD-like memory, and the administration of a PAI-1 antagonist into the hippocampus prevented this effect [138]. A shift in the balance towards proBDNF also affects synaptic plasticity: signaling of proBDNF through the p75NTR receptor promotes long-term depression, whereas that of mature BDNF through TrkB promotes LTP [133]. Thus, prenatal stress in rats reduced the activity of tPA in the hippocampus of the offspring, increased the level of proBDNF, decreased the level of mature BDNF, and impeded the induction of LTP [139]. The influence of GCs on BDNF therefore depends on the intensity and the duration of the exposure: under chronic or very intense stress, suppression of the expression of the gene and of the maturation of the protein predominates, whereas a moderate increase in GCs may, on the contrary, enhance the maturation of BDNF and the formation of memory.

The transcription factor ZBTB16 (zinc finger and BTB domain containing protein 16), the product of the *Zbtb16/Plzf* gene, performs important functions in the brain that are related to morphogenesis, cognitive abilities, and behavior, and it participates in the coordination of the stages of formation of the neocortex. In mice with knockout of *Zbtb16*, social behavior is disturbed, stereotypies appear, the propensity to risk increases, and cognitive functions suffer; in the somatosensory cortex, layer VI becomes thinner and the number of TBR1-positive neurons decreases, whereas in the prefrontal cortex the number of dendritic spines and of microglia increases and the development of oligodendrocytes and myelination are disturbed [140].

*Zbtb16* is a target of GR. In the paraventricular nucleus of the hypothalamus of adult mice of both sexes, the level of its mRNA doubled within 2 h after acute immobilization stress or after the administration of dexamethasone [141].

The GREs of the *ZBTB16* locus are located in introns [142,143]; in human cerebral organoids, dexamethasone changed DNA methylation precisely in those GREs that are located in enhancers forming loops to the transcription start site of *ZBTB16* [143]. In the same work, treatment of human cerebral organoids with dexamethasone at a concentration and for a time corresponding to clinical recommendations (100 nM, 7 days) increased the number of basal progenitors of a particular type, co-expressing *PAX6* and *EOMES*, which in species with a gyrencephalic brain contribute to the expansion of the cortex, and also the number of neurons of the upper layers; an experiment with the antagonist RU486 showed that the effect is mediated mainly by GR and not by the mineralocorticoid receptor. ZBTB16 proved to be both a sufficient and a necessary link in this effect: its overexpression in organoids reproduced the phenotype, whereas excision of exon 2 in one of the alleles by the CRISPR-Cas9 method (a complete knockout proved non-viable at the stage of induced pluripotent stem cells (iPSCs)) reduced the increase in double progenitors under the action of dexamethasone from 22.2% to a non-significant 6%. In mice, *Zbtb16* is not physiologically expressed during the period of neurogenesis, and its heterochronic overexpression in the cortex of embryos (in utero electroporation at E13.5 with analysis at E16.5) also increased the number of PAX6- and EOMES-positive progenitors [143]. In the opinion of the authors of that work, the described influence of glucocorticoids on neurogenesis is associated with favorable postnatal outcomes, and only upon exposure during the period of cortical neurogenesis, that is, before the 28th gestational week. Mendelian randomization for the enhancer variant rs648044, in carriers of the A allele of which the glucocorticoid strongly induces *ZBTB16* re-expression, revealed causal links with a higher level of education, a lower expression of neuroticism, an increase in cortical thickness, and changes in the white matter. In the prospective ITU cohort, the interaction of this allele with the mean level of maternal cortisol before the 28th week was associated with higher cognitive scores in children at the age of three years (*p* = 0.035; 246 mother and child pairs), whereas for cortisol after the 28th week, that is, after the completion of cortical neurogenesis, no such association was found (*p* = 0.46).

In humans, mutations of *ZBTB16* have been described: a heterozygous missense mutation in brothers with autism spectrum disorder and a heterozygous nonsense mutation in patients with schizophrenia, whereas the homozygous variant causes a syndrome that includes intellectual disability, microcephaly, skeletal anomalies, and hypoplasia of the external genitalia [140].

### 5.4. Genes of Inflammation and Immunity

For pro-inflammatory factors, functional regulation through GR is multilevel and combines both DNA-dependent and tethering mechanisms [36,144]. Ligand-activated GR suppresses the transcription of the key pro-inflammatory cytokines (IL-1β, IL-6, TNF-α, and also of a number of chemokines such as CCL2) through interaction with the already DNA-bound subunits p65/p50 or c-Jun/c-Fos of the transcription factors NF-κB and AP-1 and the subsequent recruitment of corepressors (GRIP1, NCoR/SMRT). These effects are supplemented by direct DNA-bound regulation. For a number of pro-inflammatory genes in immune and epithelial cells, regions have been mapped that bind GR directly and provide for the repression of these genes. Thus, activated GR suppresses the *TSLP* gene (thymic stromal lymphopoietin) through an nGRE located 850 bp upstream of the transcription start site [48]. For IL-6, no independent nGRE has been described: the DNA-binding domain of GR binds regions of the promoter that resemble the consensus GRE only moderately [145,146]. It was shown that GR regulates the expression of a subset of inflammatory genes by binding to AP-1 response elements (TRE) on DNA even in the absence of AP-1 [43]. For IL-6, the established response element is the genomic NF-κB response element (κBRE) containing a cryptic GR binding site: such elements have been found in the promoters of the genes *IL6*, *IL8*, *CCL2*, *RELB* and *ICAM1*; GR is recruited to them according to ChIP data, and mutation of its DNA-binding domain abolishes this recruitment, which is accompanied by suppression of the expression of these genes [44]. The genes *IL6*, *CCL2*, *TNF*, *IL1B*, *ICAM1*, and *RELB* are expressed in microglia, in astrocytes, and in brain endothelial cells during neuroinflammation, where they determine the spectrum of the cytokine response of the CNS. The mapping of GR binding sites for cytokine genes has been performed predominantly in peripheral tissues, and the mechanism of their regulation in the CNS still has to be elucidated in full.

There is also an inverse influence: the pro-inflammatory cytokines themselves (IL-1α, TNF-α) reduce the sensitivity of cells to glucocorticoids by disturbing the translocation of GR and by increasing the expression of the dominant negative isoform GRβ, which forms a feedback loop between inflammation and GR signaling [147].

### 5.5. Genes of Anti-Inflammatory Factors

For anti-inflammatory factors, functional regulation through GR is likewise multilevel. Ligand-bound GR induces the expression of a number of genes that suppress inflammation (for example, DUSP1/MKP-1, TNFAIP3, ZFP36), the products of which suppress MAPK and NF-κB signaling and thereby reduce the production of pro-inflammatory cytokines [62,144]. At the same time, glucocorticoids are able to induce the expression of a number of anti-inflammatory mediators [148]. Thus, GR activates the promoter of the an *ANXA* (lipocortin 1) gene in T lymphocytes [149], and dexamethasone increases the expression and the secretion of annexin A1 in rat astrocytoma cells [150]. The induction of IL-10 by glucocorticoids in human B lymphocytes is mediated not by a GRE but by the transcription factor STAT3 [151]. The influence of GCs on the IL-1 receptor antagonist (IL-1Ra) depends on the cell type: in airway epithelial cells dexamethasone induces the intracellular isoform of IL-1Ra [152], whereas in monocytes stimulated with endotoxin it suppresses its expression and secretion [153].

These DNA effects are supplemented by tethering mechanisms, in which GR, through protein–protein interaction with NF-κB, AP-1, or STAT3, changes the set of coactivators and corepressors at the promoters of the genes of anti-inflammatory factors and of their receptors, providing a fine tuning of the balance between pro-inflammatory and anti-inflammatory programs. In the CNS, the GR-mediated induction of such anti-inflammatory genes and signaling regulators in microglia and astrocytes is regarded as one of the key mechanisms that limit neuroinflammation and promote the transition of glia to a reparative phenotype.

One of the mediators of the realization of the anti-inflammatory signaling of GR is the product of the *TSC22D3* gene, the protein GILZ (glucocorticoid-induced leucine zipper) [154]. It physically binds c-Fos and c-Jun, preventing the binding of AP-1 to DNA [155], and also the p65 subunit of NF-κB [62], preventing the transfer of this factor into the nucleus [154]. *TSC22D3* undergoes transcriptional activation under the action of glucocorticoids through the interaction of GR with GRE sequences. It is assumed that in chronic hypercortisolemia a constantly elevated level of GILZ excessively suppresses immune and growth processes, whereas at a low sensitivity of GR or a low level of cortisol its deficiency opens the way to chronic neuroinflammation (no direct data on the brain were found) [156]. An association has been established between a decrease in GILZ expression and an increased risk of developing symptoms of PTSD in male mice and in men [157], whereas its lowered expression in blood is associated with a smaller volume of the hippocampus in depression [158].

DUSP1/MKP-1 “switches off” the inflammatory MAPK signaling pathways JNK and p38. Through DUSP1, GR blocks the cascades that trigger the synthesis of pro-inflammatory cytokines [106,159]. In the distal promoter region of the *DUSP1* gene (about −1.3 kb), a functional GRE has been identified; the binding of GR is accompanied by a decrease in nucleosome density, by acetylation of histones H3 and H4, and by the recruitment of the coactivator p300, which is necessary for the induction of *DUSP1* [160]. In the brain, DUSP1/MKP-1 restrains the excessive activation of JNK and p38: in neuroblastoma cells and in a primary culture of neurons, knockdown of MKP-1 enhanced the phosphorylation of JNK and cell death upon hypoxia with reoxygenation, whereas its overexpression reduced the level of phospho-JNK, the expression of pro-apoptotic genes, and cell death [161]; in mice after ischemia, MKP-1 is expressed in the nuclei of neurons and of microglia, and its knockout or pharmacological suppression increase the infarct volume and the neurological deficit [162]. However, the role of DUSP1 in the brain depends on the context: in prenatal stress, the complex of GR with the same coactivator p300 increases the level of MKP1 in the hippocampus of the offspring, which suppresses the ERK/CREB/TRKB pathway, reduces neurogenesis, and is accompanied by depression-like behavior, whereas the GR antagonist mifepristone lowered the level of MKP1 and abolished both disturbances [163].

More detailed information on the genes of pro-inflammatory and anti-inflammatory factors that are targets of GR is presented in Supplementary Tables S1 and S2.

### 5.6. Genes of Circadian Rhythms

The cellular clock is arranged as a feedback loop: the CLOCK/BMAL1 complex initiates the transcription of the PER (Period) and CRY (Cryptochrome) genes, the accumulated PER and CRY proteins suppress CLOCK/BMAL1 itself, after which they are degraded, and the cycle, about a day long, begins anew; the amount of PER in this loop is the limiting one, and therefore it is precisely this amount that sets the current phase, the internal “time” of the cell [164].

The genes *PER1* and *PER2* are targets of GR: the receptor binds GREs upstream of these genes, increasing the level of their mRNA and protein and thereby shifting the cellular clock to another phase. At the same time, in fibroblasts dexamethasone acutely induces *PER1* but not *PER2*, and the induction of *PER1* depends on functional GR. In addition, GR suppresses the transcription of the *NR1D1* gene, which encodes the auxiliary clock protein REV-ERBα, and a decrease in the level of REV-ERBα removes its inhibitory influence on the transcription of *BMAL1* [164].

In the organism there is a master oscillator in the suprachiasmatic nucleus of the hypothalamus and there are proper peripheral clocks in the cells of most tissues, which the master oscillator adjusts, in particular through the daily rhythm of glucocorticoids. Glucocorticoids that arrive outside the usual phase are able to shift the peripheral clocks separately from the master oscillator: the intake of 20 mg of hydrocortisone in the second half of the day acutely increased the expression of *PER1* in human blood mononuclear cells, and after six days the phase of *PER2*, *PER3*, and *BMAL1* in these cells shifted by 9.5 to 11.5 h, whereas the phase of the central markers, that is, of the rhythms of cortisol and melatonin, did not change, so that the periphery and the center diverged in time [165].

A disturbance of the relationship between sleep and circadian rhythms is associated with bipolar disorder [166] and with depression [167]. In post-traumatic stress disorder, changes in the daily profile of glucocorticoids and sleep disorders have been described; however, there are no data on a change in the work of the central oscillator in this condition [168].

## 6. Acute and Chronic Effects of GR Action

The severity and the persistence of the effects of GCs on the organism and on the developing brain depend not only on the properties of the object but also on the duration, the concentration, and the regimen of production or of exogenous administration of GCs. From the point of view of the duration of psychological stressful influences, acute and chronic stress are distinguished. Acute stress is the reaction of the organism to a sudden action of a stressor, characterized by a rapid engagement of adaptive mechanisms and lasting from several minutes to several hours. It includes the activation of the sympatho-adrenal-medullary (SAM) and hypothalamic–pituitary–adrenal (HPA) axes with the release of hormones such as adrenaline and cortisol in order to prepare for the fight or flight action [169,170]. Chronic stress arises upon a prolonged, continuous, or regularly repeated action of stressors over weeks or months, which leads to failures in the work of the HPA axis and increases the risk of mental and other diseases [171].

Traumatic stress is singled out in particular; it arises after events that threaten life (for example, an attack or a catastrophe). Traumatic stress differs in that it has deeper psychological consequences, is characterized by intrusive memories and flashbacks, and may lead to the development of PTSD [172,173,174].

Numerous studies of the consequences of both acute and chronic stress, as well as of prenatal exposure to GCs during pregnancy in humans and in animals, have recorded undesirable changes in neurogenesis and neurotoxic effects [175,176,177], and an increased probability of emotional and cognitive problems and of other dysfunctions in the work of the central nervous system in the offspring [177,178,179,180,181].

However, according to a number of data, some of the negative changes caused by stress or by an excess of GCs in adults and in adolescents are temporary. Thus, in adult rats a single acute stress reduced the number of new granule cells in the dentate gyrus; this effect depended on the stress-induced rise in the level of corticosterone and was abolished when this rise was prevented, and after 3 weeks the difference from the control disappeared, most probably, in the opinion of the authors, owing to a lower death rate of the new cells in the animals that had undergone stress [182]. In rodents subjected to stress in adolescence, the decrease in cell proliferation in the hippocampus and the depression-like behavior disappeared in a number of works after a period without stress, at least in stress-resistant animals, whereas in other studies the cognitive impairments persisted or even appeared for the first time in adulthood. The decrease in neurogenesis and of neuroplasticity in the hippocampus, the cognitive impairments, and the depression-like behavior caused by such stress could be prevented or eliminated with the help of antidepressants, of inhibitors of glutamate receptors, of GR antagonists, or of a diet enriched with omega-3 fatty acids and vitamin A [183].

A number of works have shown that stress acts on the neuronal lineage, reducing the number of new granule cells through suppression of proliferation [182,184,185] or through a decrease in their survival [186]. High concentrations of corticosterone cause apoptosis of hippocampal cells [184,187,188,189] and of neuron-like PC12 cells [190], which is at least in part mediated by excessive activation of GR [187]. In addition, glucocorticoids suppress the proliferation of glial progenitor cells: in adult rats, prolonged administration of corticosterone sharply reduced the proliferation of NG2+ oligodendrocyte progenitor cells in the white and gray matter of the brain [191], and in the hippocampus it reduced the formation of new NG2+ cells, of microglial cells, and of oligodendrocytes [192]. Corticosterone, apparently through GR, also reduces the expression of the astrocyte marker GFAP in the hippocampus and in the cortex, and after adrenalectomy the expression of *GFAfap* increases [193]. At the same time, physiological signaling through GR is necessary for the normal development of oligodendroglia: in mice with deletion of GR in postnatal oligodendrocyte progenitor cells, the density of oligodendrocytes and of myelinated axons in the hippocampus decreased, and in adulthood long-term potentiation and the formation of memory were disturbed [194]. In addition, chronic exposure to exogenous glucocorticoids leads to immune priming and to hyperreactivity of microglia [195].

At the same time, some studies indicate an absence of effect or even a beneficial influence of acute stress [196,197]. Thus, in the work of Kirby et al. [198], out of three models of acute stress only a three-hour immobilization significantly enhanced the proliferation of progenitor cells in the dentate gyrus, whereas a novel environment and electric skin stimulation produced no effect; the administration of corticosterone reproduced the effect only at a high dose. It is mediated by fibroblast growth factor 2 (FGF2) secreted by astrocytes. After two weeks, but not after two days, the same animals showed an improved memory of fear extinction, which coincided in time with an increased activation of newborn neurons.

Another mechanism of the beneficial action of acute stress has been described in the prefrontal cortex. Behavioral stress or a short-term action of corticosterone causes there a delayed and persistent enhancement of the synaptic response and of the surface expression of NMDA and AMPA receptors in pyramidal neurons through the induction of the kinase SGK and the activation of Rab4, which provides for the return of the receptors from early endosomes to the membrane; working memory is thereby improved in an SGK-dependent manner [199,200]. This effect belongs to the genomic ones: it is abolished by GR antagonists and by inhibitors of transcription and translation. Alongside it, a rapid non-genomic mechanism operates: corticosterone, acting through membrane GRs, increases the number of presynaptic SNARE complexes that provide for the fusion of vesicles with the presynaptic membrane, that is, it expands the pool of glutamate vesicles ready for release [201].

Most probably, it is impossible to give an unambiguous assessment of the consequences of acute stress, in particular for the functioning of the hippocampus, since in this case its character, including the intensity, the duration, and the period of life at which the exposure occurs, is probably of importance [196,202].

E. Ronald de Kloet and co-authors proposed the concept of the receptor balance of mineralocorticoid (MR) and glucocorticoid (GR) receptors (MR:GR), which explains how one and the same hormonal action can be both adaptive and damaging for neurons [28]. According to this concept, at a basal level of corticosterone the high-affinity MRs in the hippocampus are already almost fully occupied, whereas during stress, when the concentration of the hormone increases, the low-affinity GRs are activated in addition [28]. Since the nuclear MRs are already occupied at a basal level of the hormone, the rapid effects of corticosterone in the initial phase of the stress reaction are provided by membrane-associated MRs, whose affinity for corticosterone is lower than that of the nuclear form [32,203,204]. Activation of MR in limbic structures promotes selective attention, the appraisal of the situation, and the choice of a behavioral strategy, and also increases the excitability of hippocampal neurons, whereas the slower GR-mediated effects suppress this temporarily increased excitability and provide for the consolidation of the stressful experience in memory, the termination of the stress reaction, and the restoration of homeostasis [32,203,205].

Unlike a healthy adaptive reaction, in chronic stress a dysregulation of the system occurs: against the background of a decrease in the function or in the expression of MRs, an excessive and prolonged activation of GR-dependent signaling pathways develops [26]. This leads to a disturbance of the mechanisms of glucocorticoid-mediated negative feedback in the hypothalamic–pituitary–adrenal axis and to the development of glucocorticoid resistance [116]. In addition, a chronic “overload” of GRs creates conditions for neurotoxicity. Glucocorticoids suppress glucose uptake in cultures of hippocampal neurons and glia by 20 to 33%; the effect develops not earlier than after 4 h, is abolished by an antagonist of type II but not of type I receptors, and is not reproduced in cultures of the hypothalamus, of the cortex, and of the cerebellum, despite the presence of corticosteroid receptors in them [206]. In hippocampal astrocytes, glucocorticoids, in addition to suppressing glucose transport, reduce the affinity of glutamate uptake and worsen the survival of the cells under hypoglycemia, thereby weakening the ability of astrocytes to remove damaging glutamate from the synapse [207]. Changes in glutamate release, of the activity of its receptors, and of its clearance increase the vulnerability of neurons to excitotoxic damage [201]. A prolonged excess of glucocorticoids is associated with atrophy of the hippocampus in Cushing’s syndrome, in severe depression, and in PTSD, although the causal sequence remains unclear [208].

A number of synthetic GCs differ from the natural ones in their pharmacokinetics and in their affinity for the GR and MRs. In particular, dexamethasone, unlike the natural GCs, binds weakly to transcortin (the natural carrier of glucocorticoids in blood serum) and possesses a considerably higher affinity for GR than for MR. By activating GRs in the corticotrophs of the anterior lobe of the pituitary, dexamethasone imitates the negative feedback signal of the HPA axis and suppresses the secretion of ACTH. For this it does not need to penetrate the blood–brain barrier, since the anterior lobe of the pituitary is located outside it [209,210]. A decrease in the level of ACTH, in its turn, leads to a decrease in the endogenous secretion of cortisol or corticosterone by the adrenal cortex [211,212]. At the same time, dexamethasone itself, being a substrate of the P-glycoprotein of the blood–brain barrier, penetrates poorly into the brain and does not compensate for the arising deficiency of endogenous GCs [210]. Thus, the MRs in the brain, which normally are already occupied at a basal level of GCs, remain unoccupied and cannot regulate their target genes, while in peripheral tissues and in the pituitary the synthetic GCs continue to act through GRs [213]. In addition, MR and GR are able to form heteromeric complexes that influence the transcriptional response [214]: MR within such complexes weakens the binding of GR to DNA, and different therapeutic glucocorticoids induce the interaction of GR with MR to different degrees [33]. An insufficient activation of MR in the brain may underlie the psychological side effects of therapy with dexamethasone; thus, in children with acute lymphoblastic leukemia who received dexamethasone, severe psychological disturbances and sleep disturbances developed in approximately 30% of cases, and these were alleviated by the addition of low doses of cortisol, which restores the occupancy of MR [213]. According to the concept of the MR:GR balance, a stressful influence at the moment of an imbalance of MR and GR signaling may increase the probability of undesirable cognitive and psychological consequences [28,32].

The absence of the context of MR effects should also be taken into account when acute stress is modeled with pharmacological GCs. In this case a substantial difference from the natural stress response is possible. Pharmacological modeling does not fully reproduce the complex reaction of the organism to stress, also because there is an absence not only of the early phase of MR effects but also of the involvement of the sympatho-adrenomedullary (SAM) axis in the very first seconds after the stressful influence, as well as of the engagement of additional regulatory systems such as endocannabinoid [215] and opioid signaling [170,216,217].

The use of exogenous GCs is more justified when chronic stress is modeled, when a constantly high level of the hormone disturbs the negative feedback of the HPA axis, which is characteristic of deep depressive states. Thus, a widely used method is the administration of corticosterone (CORT) to rodents: it reproduces mood disorders and is accompanied by dysfunction of the HPA axis [184]. This method makes it possible to study the direct contribution of glucocorticoids to the pathogenesis of depression and anxiety, separating their effects from physical pain or other factors. However, in this case too it should be taken into account that this model imitates only the endocrine finale of the stress reaction, omitting other aspects of the real experience of a stressful situation.

As regards the reaction of the immune system of the brain to stress, the anti-inflammatory action of GCs is considered classical: through genomic mechanisms, including transrepression of NF-κB and AP-1, they suppress the expression of pro-inflammatory cytokines [218,219]; in addition, cytosolic GR is able to bind the subunits of NF-κB and IκB [102]. However, the view of GCs as universally anti-inflammatory hormones has been revised: in the CNS they may also exert a pro-inflammatory action [220]. The key factor here turns out to be the timing of the exposure relative to the immune challenge: if the inflammatory stimulus precedes the stress, the stress has an anti-inflammatory action, whereas stress that precedes the stimulus enhances the subsequent pro-inflammatory reaction [221]. Thus, a single acute inescapable stress in male rats led, 24 h later, to an enhanced expression of IL-1β and IL-6 in hippocampal microglia in response to lipopolysaccharide, that is, to an immune priming of microglia; this effect was blocked by the GR antagonist RU486 and by adrenalectomy [221]. Chronic administration of corticosterone also caused priming of microglia and, in addition, increased the expression of NLRP3 and of the microglial activation markers Iba-1 and MHCII in the hippocampus [195]. In this state, microglial cells switch to a sensitized mode, which is characterized by morphological changes [222] and by epigenetic reprogramming, that is, by long-term changes in epigenetic marks in the enhancers of the genes of the inflammatory response, shown for the priming of microglia by inflammatory stimuli and in aging [223,224,225]. Upon a subsequent immune or psychogenic challenge, such primed cells display hyperreactivity, secreting an excessive amount of pro-inflammatory and neurotoxic factors, which transforms the adaptive stress response into pathological neuroinflammation [222,226]. Thus, both an acute and a chronic increase in GCs preceding an immune challenge may sensitize microglia and thereby potentially increase the vulnerability of the brain to neurodegenerative and mental diseases.

Thus, on the one hand, acute stress or a short-term increase in glucocorticoids promotes the suppression of excessive inflammation, whereas on the other hand, acute stress priming of microglia increases its sensitivity to subsequent immune challenges. At the same time, chronic hypercortisolemia forms a persistent hyperreactivity of microglia and a pro-inflammatory environment that increases the vulnerability of the brain to neurodegenerative and mental diseases.

It is important to emphasize that different models of chronic stress and different regimens of glucocorticoid administration yield not simply a “different strength of effect” but qualitatively different pictures of behavior, of molecular changes, and of changes in the structure of the brain. This is related to the type of stressor, to its predictability and controllability, to its duration, and, in GC models, also to the protocol of glucocorticoid use (pulsatile or constant, the dose, the drug). Thus, the study of McLaughlin et al. [227] examined the influence of chronic restraint stress on male rats depending on the number of hours per day and on the total number of days. The results showed that only in rats subjected to restraint for 6 h a day for 21 days were there retraction of the apical dendrites in the hippocampus, persistent disturbances of spatial memory, and a reduced weight gain. In the work of Li et al. [228], rats were subjected to chronic unpredictable mild stress lasting 3 days, 2 weeks, and 6 weeks. Transcriptomic analysis of the hippocampus revealed 501, 442, and 235 differentially expressed genes, respectively. Moreover, the direction of the regulation patterns of the 3-day and of the 6-week stress was opposite. Gene ontology analysis revealed that the early stages of stress are accompanied by changes in the genes of the ECM and of repair, whereas the late stages are accompanied by a shift towards apoptosis and senescence. The authors conclude that at the early and middle stages of chronic stress the adaptive processes of regulation and of recovery after damage predominate, whereas at the late stages the processes of damage, namely apoptosis and senescence, predominate in the hippocampus.

Curious results were obtained in the work of Liu et al. [229], in which the consequences of exposure to different kinds of stress were studied in rats: physical (chronic unpredictable mild stress, learned helplessness) and psychological (chronic restraint stress and social defeat stress). Although pronounced depression-like behavior of the animals was revealed for all four models, bioinformatic analysis showed that the molecular changes in the hippocampus caused by physical and by psychological stress differ radically (nine common metabolites changed in opposite directions). This indicates that one and the same mental disorder (in this case a depression-like one) may be based on different molecular mechanisms.

It has been confirmed experimentally that a different regimen of GC administration (constant or pulsatile) also leads to different patterns of gene regulation and to different neuroendocrine and behavioral reactions [87,230,231]. A cross-sectional MRI study of patients aged 9 to 18 years with Duchenne muscular dystrophy revealed differences in the influence on the brain of a daily and of an intermittent (10 days of intake, 10 days of interruption) intake of GCs [232]. Changes in gray matter volume in individual brain areas in comparison with healthy peers were found in both groups; however, with daily intake they affected more areas and were more pronounced; in addition, only in that group did the total volumes of gray and white matter and of cerebrospinal fluid differ from the control. The authors relate this to the fact that with daily intake the GR-mediated processes are constantly hyperactivated, whereas the MR-mediated ones are relatively underactivated against the background of chronic suppression of endogenous cortisol (see above on the concept of the MR:GR balance and on the action of synthetic GCs), whereas with the intermittent regimen the own secretion of cortisol is restored during the intervals [232]. It should be taken into account, however, that the groups received different drugs (deflazacort and prednisone, respectively) and were examined in different centers, and therefore the conclusion about the influence of the regimen of intake itself is of a preliminary character.

Differences in stressful influences are also reflected in different epigenetic changes in brain tissues [233,234]. Thus, chronic stress often leads to persistent changes in the epigenome, whereas acute stress causes changes that may be temporary. In particular, one of the epigenetic changes that are significantly unfavorable for mental health is a persistent increase in the methylation of the GR gene *NR3C1* and a decrease in the methylation of the *FKBP5* gene, which disturbs the self-regulation of stress [116].

Epigenetic changes have also been described in traumatic stress, and they are related not to the fact of the trauma itself but to the development of the disorder: in a meta-analysis of 23 cohorts, people with PTSD differed from those who had experienced trauma but had not developed PTSD in the methylation of a number of CpG sites in blood; for many of these sites a correlation of the levels of methylation between blood and brain has been shown, as well as an association with PTSD in several areas of the brain [235]. However, the directions and the magnitude of the methylation of one and the same gene in chronic and in traumatic stress may not coincide. In particular, chronic maltreatment of children is associated with hypermethylation of *NR3C1* and with more pronounced depressive symptoms [236], whereas in patients with PTSD, on the contrary, hypomethylation of the *NR3C1* promoter is detected [237]. In addition, a genome-wide study of DNA methylation in peripheral blood in people with PTSD and with depression revealed no association significant at the genome-wide level (FDR < 0.05) for either of the disorders. Nevertheless, the genes with differential methylation at the level of suggestive significance in these two disorders for the most part did not overlap, and different biological pathways turned out to be enriched: in PTSD, the regulation of synaptic plasticity, oxytocin signaling, the cholinergic synapse, and the pathways of inflammatory diseases; in depression, phosphatidylinositol signaling and the cell cycle [238].

It should also be noted that stress may affect representatives of one and the same group differently [239]. Individual differences in the consequences of stress are based on genetic predisposition in the form of features of the nucleotide sequence of DNA and of inherited or acquired epigenetic marks. Genetic predisposition manifests itself through polymorphisms in the genes encoding components of the stress system (for example, glucocorticoid receptors), which influences the sensitivity of the organism to stress factors. Epigenetic mechanisms, including DNA methylation, histone modification, and microRNAs, form an additional level of regulation that can both enhance and weaken the effects of genetic variations. Of particular significance are epigenetic changes in the promoter regions of the key genes of the stress system (*NR3C1*, *FKBP5*, *BDNF*), which, according to some data, may be transmitted to subsequent generations, although the mechanisms of transgenerational epigenetic inheritance remain a subject of investigation. Thus, it is precisely the combination of genetic and epigenetic factors that creates the unique profile of stress resistance of each individual, and this is of fundamental importance for understanding the mechanisms of individual vulnerability and resistance to stressful influences [116].

Biological sex is another important source of individual variability of the response to stress: sexual dimorphism may increase the risk of developing stress-related disorders, including PTSD, to which women are more vulnerable. At the same time, in many frequently used animal models of stress, sex differences are not taken into account [240]. However, this topic is very extensive and requires separate consideration.

## 7. Conclusions

The glucocorticoid receptor is a multilevel integrator of hormonal, metabolic, and stress-dependent signals, capable of finely readjusting the transcriptional programs of the cell depending on the context. This is manifested at several levels, the first of which is the diversity of motifs in the binding sites of GR, and also, apparently, the ambiguity of predicting the outcome of transcription from the presence of a particular motif without taking the context into account, as is characteristic of the nGRE. Thus, one and the same gene under an identical hormonal load may be activated in one cell type and repressed in another, and one and the same group of common target genes may display regulation in opposite directions even in two related cell types.

The second level of regulation by GR is that it does not simply use the existing epigenetic landscape; it actively rebuilds it, influencing the activity of DNMT3a/b, of DnmNMT and of the TET dioxygenases. It is particularly significant that through a TET3-dependent mechanism these changes are able to be transmitted to daughter cells that have never been in contact with glucocorticoids, thereby creating a molecular basis for a long-term change in the genetic program. An example of this is the persistent demethylation of *FKBP5*, maintained through a decrease in DnmNMT and a loss of MeCP2 binding, which leads to insensitivity to GCs and to the development of hypercortisolemia.

In addition to its direct influence on the epigenome, GR, acting on signaling cascades (in particular, pERK1/2→MSK1→H3S10p-K14ac), provides for the preparation of chromatin for the consolidation of the stressful experience in memory within tens of minutes, that is, even before the beginning of the transcriptional activity of the receptor as a transcription factor. This allows one to take a new look at the distinction between the “genomic” and the “non-genomic” effects of GR, which is to a considerable extent conventional, since in practice they form a continuous cascade in which rapid cytoplasmic events predetermine the subsequent nuclear ones.

The clinical and experimental data presented here emphasize the complex nature of glucocorticoid signaling. On the one hand, physiological and therapeutic doses of glucocorticoids are necessary for the normal maturation of organs and systems, for the realization of circadian rhythms, for the maintenance of metabolic and immune homeostasis, and for the treatment of a number of diseases. On the other hand, an excess of endogenous or exogenous glucocorticoids in critical windows of development, as well as their chronic or traumatic increase in adulthood, may contribute to the formation of cognitive, affective, and neurodegenerative disorders.

An understanding of which transcriptional and epigenetic programs are activated by GR depending on the type of cell, on age, on the model of stress, and on the character of the hormonal influence opens the way to a more selective therapy in the future. Promising strategies appear to be those directed at selective modulation of the MR/GR balance; intervention in the assembly of particular coregulatory complexes (for example, NCoR/SMRT and HDAC3); targeting of the DNMT, TET, and HDAC cascades in critical time windows; and also the development of drugs capable of influencing the GR-dependent formation of epigenetic memory without a crude suppression of the whole glucocorticoid axis.

Despite considerable progress in the understanding of GR signaling, a number of fundamentally important unstudied aspects of the mechanisms of GR action remain, and they limit the possibility of developing targeted therapeutic interventions. In particular, it has not been fully determined which configurations of GR and coregulatory complexes are assembled and at which moment for the activation of “useful” or undesirable effects at the level of individual populations of neurons and glial cells. There is a critical lack of information on the construction of the chains of molecular cascades and on the mechanisms of the influence of GR on the epigenetic modifiers TETs and HDACs (and other effector molecules), as well as on the features and the variants of these pathways depending on the type of cells and tissues. Finally, the question remains open whether it is possible to selectively modulate individual branches of GR signaling by the type of elements (GRE/nGRE, composite sites), by coupling with AP-1, NF-κB, or Stat3, or through a targeted intervention in the DNMT, TET, and HDAC cascades, without a crude suppression of the whole HPA axis. The answers to these questions are critical for the creation of drugs capable of selectively readjusting GR-dependent programs in the required cells and time windows, minimizing the risk of systemic side effects.

## Figures and Tables

**Table 1:** Comparative analysis of the distribution and function of MR and GRs in the brain.

Parameter	Glucocorticoid Receptor (GR)	Mineralocorticoid Receptor (MR)	What Is Important for Interpretation
Affinity	Low (Kd about 5 nM for corticosterone). It is activated in addition to MR only at high levels of the hormone, that is, at the circadian peak and during stress [28].	High (Kd about 0.5 nM, approximately 10 times higher than that of GR). It is substantially occupied by corticosterone already at basal levels [28].	As the level of the hormone rises, MR is engaged first and GR afterwards. This helps to explain why low and high levels of the hormones may trigger different programs of gene expression.
Localization in the brain	Broadly expressed; the highest levels are in the structures that regulate stress: the paraventricular nucleus of the hypothalamus, the network of the prefrontal cortex, hippocampus, and amygdala, and the ascending aminergic systems. In the hippocampus, GR mRNA is low in field CA2 and moderate in CA1 [31,32].	The highest levels are in the limbic structures: the hippocampus (the dentate gyrus and the CA fields, with the highest expression in CA2), the lateral septum, and the amygdala; to a lesser extent, the nuclei of the thalamus and of the brainstem [28,31,32].	Brain areas with different densities of the receptors will respond differently to stress and to circadian fluctuations of the hormones.
Cell types in the brain	All cell types: neurons, astrocytes, oligodendrocytes, microglia, and also the endothelium of the brain vessels [32,36].	Predominantly neurons (glutamatergic and, to a lesser extent, GABAergic) and astrocytes; in oligodendrocytes and microglia it is not detected according to single-cell sequencing data [31].	The expression of MR and GR depends on the cell type. Therefore, when analyzing the responses mediated by MR and GR, one cannot focus on neurons alone, since the contribution of glia and microglia may be critically important [34].
Mechanism of binding to DNA	Through GREs, through negative GREs (the nGRE, which of all the steroid receptors only GR is able to use), and through composite elements next to other transcription factors [35].	Predominantly through GREs: in the hippocampus the MR binding sites are almost exclusively GRE-like, and specificity is provided by the neighboring motif of the NeuroD factors [35].	Specificity is often determined not by the receptor itself alone but also by the accessibility of chromatin and by the neighboring transcription factors [35,36].
Role in pathology	Hyperactivation is associated with neurodegeneration and with cognitive impairments [31,32].	Carriage of a functional MR haplotype with an increased activity of the receptor is associated with a lower risk of depression, predominantly in women [32].	The direction of the effect is set not by one receptor but by the ratio of the activation of MR and GR, and therefore a disturbance of their balance is very important [32].
Key targets	*CRH* in the hypothalamus and *POMC* in the pituitary (repression, presumably through an nGRE); *AQP4* in astrocytes: its expression is suppressed in a model of chronic excess of glucocorticoids, yet the participation of GR itself in this has not been shown, and the authors explicitly call the mechanism undetermined [31].	*Jdp2* is a hippocampus-specific target of MR: the locus binds MR, and in mice with knockout of MR in the forebrain the level of its mRNA is reduced [31,35].	One gene does not always explain the effect; the coordinated work of whole sets of genes is often important [31].
Common targets	*FKBP5*, *SGK1*, *GILZ*, and *PER1* are regulated by both receptors [35,36].		It is precisely these genes that are usually used as indicators of the action of glucocorticoids, but their expression does not allow one to judge which of the two receptors has acted [35].

**Table 2:** Comparison of four types of GREs: classical, negative (nGRE), composite, and semi-palindromic.

Type of Element	Classic (+) GRE	Negative GRE (IR nGRE)	Composite Element	GRE Half-Site
Main function	Activation of transcription: agonist-bound GR recruits the coactivators SRC1, SRC2/TIF2, and SRC3 [38].	Direct repression of transcription by agonist-bound GR [38].	Integration of the GR signal with the signal of another transcription factor; the outcome may be either activation or repression [42].	Repression of inflammatory genes upon the binding of GR to a half-site hidden within the motif of another factor [43,44].
Sequence structure	An imperfect palindrome: two inverted hexameric half-sites separated by three nucleotides, with the consensus 5′-AGAACAnnnTGTTCT-3′; real sites deviate from the consensus, for example the GRE of the TAT gene, 5′-TGTACAnnnTGTTCT-3′ [38,42].	An inverted repeat of the motifs CTCC and GGAGA separated by 0, 1, or 2 nucleotides (IR0, IR1, IR2); it is not similar to the classic GRE [38].	A GR binding site next to the site of another factor (AP-1, NF-κB, STAT, and so on); a particular case is the tethering element, where GR has no site of its own [42].	A single AGAACA hexamer; structurally, the mode of binding has not been characterized. Such half-sites have been found within κB elements and within the AP-1 motif TRE [42,43].
Type of GR binding	A dimer in the head-to-head orientation; the contacts of the D loops stabilize the dimer and create positive cooperativity [42].	Two monomers at non-identical everted sites in the head-to-tail orientation, rotated by 180 degrees: dimerization is impossible, and binding proceeds with strong negative cooperativity [48]. The affinity of GR for the nGRE is lower than for the classic GRE [38]. Among the steroid receptors, only GR is able to bind such elements [44].	A GR dimer at its own site; in tethering, GR does not contact DNA and is retained by protein-protein contacts [42].	A monomer; direct binding to DNA is obligatory, since a GR mutant with disturbed DNA recognition (K442A/R447A) loses the ability to repress [44].
Interaction with other transcription factors	Not obligatory, GR acts autonomously.	Not obligatory, GR acts autonomously.	Obligatory: it is the partner factor that sets the outcome.	Not obligatory but possible; an enrichment in particular motifs next to the half-sites has not been shown [42].
Cofactors	The coactivators SRC1, SRC2/TIF2, SRC3, and CBP/p300 [38].	The corepressors SMRT and NCoR, which recruit HDAC2 and HDAC3 [38].	A common set of cofactors of GR and of the partner factor; in macrophages, repression proceeds through the corepressor function of GRIP1 [41].	Not specifically studied; for the repression of κB-dependent genes, the binding of GR to DNA is by itself sufficient [44].
Effect on chromatin	An increase in chromatin accessibility and activation of transcription; GR predominantly occupies regions that are already open, but its binding additionally increases the accessibility [42,49].	Deacetylation of histones owing to the recruitment of HDAC2 and HDAC3 as part of a complex with SMRT and NCoR [38].	It depends on the partner factor; in the regions of GR-dependent repression the level of histone acetylation is decreased [41].	Not specifically studied.
Examples of regulated genes	*TAT*, *GPX3*; in the brain, *FKBP5*, *SGK1*, and *PER1* [35,38].	*TSLP*: in mice with knockout of GR in keratinocytes the agonist no longer suppresses *Tslp*, and the antagonist RU486 prevents the assembly of the repressing complex. In total, about a thousand orthologous genes with conserved IR nGREs have been found in the mouse and human genomes, and their suppression in vivo depends on GR [38]. In the brain, the repression of *CRH* and *POMC* is assigned to this type, although for POMC it has been noted that in a number of contexts repression is provided not by the nGRE itself [50].	The human *CRH* gene, where repression is provided by composite regulation [51]; the *POMC* promoter, where the outcome depends on the way in which GR is recruited by the factor STAT3 [45].	*IL8*, *CCL2*, *RELB*, *PLAU*, and *ICAM1* through half-sites within κB elements [44]; *IL6*, *IL11*, *VCAM1*, *CSF1*, *IL1A*, and *MMP13* through a half-site within the AP-1 motif [43].
Dependence on context	Moderate: it depends on the availability of coactivators and on the state of chromatin.	High: the complex is assembled only on agonist-bound GR, and the antagonist RU486 prevents its formation [38].	Very high: it is determined by the presence and the activity of the partner factor, by the cell type, and by the signaling environment.	High: repression is manifested only at a part of the inflammatory genes [43].
Role in the action of GR	Activation of metabolic and adaptive programs.	Direct suppression of genes, including those with which the side effects of glucocorticoid therapy are associated [38].	Fine tuning: one and the same signal through GR gives a different outcome depending on the partner.	It explains a part of the anti-inflammatory action that was previously attributed entirely to tethering [43,44].

The assignment of a site to one type or another does not predict the direction of the effect: in macrophages, GR both activates and represses genes at all three types of region, that is, at tethering sites, at GREs, and at GRIP1-bound elements, and the outcome is determined by the epigenetic state and by the context [41].

## Data Availability

No new data were created or analyzed in this study. Data sharing is not applicable to this article.

## Supplementary Materials

The following supporting information can be downloaded at: https://www.mdpi.com/article/10.3390/ijms27209027/s1. References [241,242,243,244,245,246,247,248,249,250,251,252,253,254,255,256,257,258] are cited in Supplementary Materials.

## Abbreviations

The following abbreviations are used in this manuscript:

11β-HSD2	11β-hydroxysteroid dehydrogenase type 2
A549	human lung adenocarcinoma cell line
acetyl-CoA	acetyl coenzyme A
ACTH	adrenocorticotropic hormone
AMPA	α-amino-3-hydroxy-5-methyl-4-isoxazolepropionic acid
Anx-A1	annexin A1
AP-1	activator protein 1
ATAC-seq	assay for transposase-accessible chromatin sequencing
ATP	adenosine triphosphate
AtT-20	mouse pituitary corticotroph adenoma cell line
BDNF	brain-derived neurotrophic factor
BMAL1	brain and muscle ARNT-like 1
BRG1	brahma-related gene 1
cAMP	cyclic adenosine monophosphate
CBP	CREB-binding protein
CCL2	C-C motif chemokine ligand 2
ChIP	chromatin immunoprecipitation
ChIP-seq	chromatin immunoprecipitation sequencing
CLOCK	circadian locomotor output cycles kaput
CNS	central nervous system
CORT	corticosterone
COX-2	cyclooxygenase 2
CpG	cytosine–phosphate–guanine dinucleotide
cPLA2	cytosolic phospholipase A2
CREB	cAMP response element-binding protein
CRH	corticotropin-releasing hormone
CRISPR-Cas9	clustered regularly interspaced short palindromic repeats/CRISPR-associated protein 9
CRY	cryptochrome
DNA	deoxyribonucleic acid
DNMT1	DNA methyltransferase 1
DNMT3a	DNA methyltransferase 3a
DNMT3b	DNA methyltransferase 3b
DUSP1	dual specificity phosphatase 1
ECM	extracellular matrix
EGF	epidermal growth factor
EGR1	early growth response protein 1
Ehmt2 (G9a)	euchromatic histone lysine methyltransferase 2
Elk-1	ETS domain-containing protein Elk-1
EOMES	eomesodermin
ERK1/2	extracellular signal-regulated kinases 1/2
FDR	false discovery rate
FGF2	fibroblast growth factor 2
FKBP5	FK506-binding protein 5
FKBP51	FK506-binding protein 51
GC, GCs	glucocorticoid, glucocorticoids
GFAP	glial fibrillary acidic protein
GILZ	glucocorticoid-induced leucine zipper
GPCR	G protein-coupled receptor
GRα, GRβ, GRγ	glucocorticoid receptor isoforms alpha, beta, gamma
GRE	glucocorticoid response element
GR, GRs	glucocorticoid receptor, glucocorticoid receptors
GRIP1	glucocorticoid receptor-interacting protein 1
H3S10p-K14ac	histone H3 serine 10 phosphorylation and lysine 14 acetylation
HDAC	histone deacetylase
HEK293T	human embryonic kidney cell line
HPA	hypothalamic–pituitary–adrenal axis
Hsc70	heat shock cognate protein 70
HSP	heat shock protein
HSP90	heat shock protein 90
HT-22	mouse hippocampal neuronal cell line
Iba-1	ionized calcium-binding adapter molecule 1
ICAM1	intercellular adhesion molecule 1
IκB	inhibitor of κB
IL-10	interleukin 10
IL-1α	interleukin 1 alpha
IL-1β	interleukin 1 beta
IL-1Ra	interleukin-1 receptor antagonist
IL-6	interleukin 6
IL8	interleukin 8, also CXCL8
iNOS	inducible nitric oxide synthase, NOS2
iPSCs	induced pluripotent stem cells
IRAK3	interleukin-1 receptor-associated kinase 3, IRAK-M
ITU	InTraUterine sampling in early pregnancy
JNK	c-Jun N-terminal kinase
κBRE	κB response element
LTP	long-term potentiation
MAPK	mitogen-activated protein kinase
MDD	major depressive disorder
MeCP2	methyl-CpG-binding protein 2
mGR	membrane glucocorticoid receptor
MHCII	major histocompatibility complex class II
MKP-1	MAP kinase phosphatase 1
MR, MRs	mineralocorticoid receptor, mineralocorticoid receptors
mRNA	messenger RNA
MSK1/2	mitogen- and stress-activated kinases 1/2
NCOA1, NCOA2, NCOA3	nuclear receptor coactivators 1, 2, 3
NCoR1, NCoR2	nuclear receptor corepressors 1, 2
Nedd4	neural precursor cell-expressed developmentally downregulated 4
NF-κB	nuclear factor kappa B
NG2	neuron-glial antigen 2, CSPG4
nGRE	negative glucocorticoid response element
NLRP3	NOD-like receptor protein 3
NMDA	N-methyl-D-aspartate
NMDAR	NMDA receptor
NO	nitric oxide
NR1D1	nuclear receptor subfamily 1 group D member 1
NR2A, NR2B	NMDA receptor subunits 2A, 2B
NR3C1	nuclear receptor subfamily 3 group C member 1
NurRE	Nur response element
p300	E1A-binding protein p300
p75NTR	p75 neurotrophin receptor
PAI-1	plasminogen activator inhibitor-1
PAX6	paired box protein 6
PBX3	pre-B-cell leukemia homeobox 3
PC12	rat adrenal pheochromocytoma cell line
pCAF	p300/CBP-associated factor
PCR	polymerase chain reaction
PER1, PER2, PER3	period circadian proteins 1, 2, 3
PI3K	phosphoinositide 3-kinase
PLCγ	phospholipase C gamma
POMC	pro-opiomelanocortin
proBDNF	precursor of BDNF
PTSD	post-traumatic stress disorder
Rab4	Ras-related protein Rab-4
RELB	RELB proto-oncogene, NF-κB subunit
REV-ERBα	reverse erythroblastosis virus alpha
RNA	ribonucleic acid
RNA-seq	RNA sequencing
RU486	mifepristone, glucocorticoid receptor antagonist
SAM	sympatho-adreno-medullary axis
scATAC-seq	single-cell ATAC sequencing
scRNA-seq	single-cell RNA sequencing
SERPINE1	serpin family E member 1
SGK1	serum/glucocorticoid regulated kinase 1
Shp2	Src homology 2 domain-containing phosphatase 2
SMRT	silencing mediator for retinoid and thyroid hormone receptors
SNARE	soluble NSF attachment protein receptor
sPLA2	secretory phospholipase A2
Src	non-receptor tyrosine kinase Src
SRC-1, SRC-2, SRC-3	steroid receptor coactivators 1, 2, 3
STAT3	signal transducer and activator of transcription 3
SWI/SNF	switch/sucrose non-fermentable
TET1, TET2, TET3	ten-eleven translocation methylcytosine dioxygenases 1, 2, 3
TF, TFs	transcription factor, transcription factors
TNF-α	tumor necrosis factor alpha
TNFAIP3	TNF alpha-induced protein 3
tPA	tissue plasminogen activator
TRE	TPA response element
TrkB	tropomyosin-related kinase B
TSC22D3	TSC22 domain family member 3
TSLP	thymic stromal lymphopoietin
TSS	transcription start site
U937	human promonocytic cell line
UHRF1	ubiquitin-like with PHD and RING finger domains 1
ZBTB16	zinc finger and BTB domain containing 16
ZFP36	zinc finger protein 36
