# Supplementary material for: Architect or Destroyer: The Diversity of Glucocorticoid Receptor Signaling Pathways in the Brain
Source: Int J Mol Sci. 2026 Oct 12;27(20):9027. doi: 10.3390/ijms27209027 (PMC13704554; doi:10.3390/ijms27209027)
Supplement: Supplementary file 1 [file ijms-27-09027-s001.zip › ijms-4554531-supplementary.pdf]

Supplementary Materials

Table S1. Pro-inflammatory factors: mechanisms of their regulation by the glucocorticoid receptor

Gene	Type	Mechanisms of regulation through GR	Data on the CNS	Comment
IL6	Pro-inflammatory cytokine	GRE-like motifs have been revealed in the IL6 promoter that overlap the enhancer, the TATA box, and the region of the transcription start, and the GR-dependent repression is explained by their occlusion [145]. In RAW 264.7 macrophages, five potential GR binding sites have been predicted, and mutation of the second of them changes the basal and the LPS-inducible activity of the promoter [146]. GR also binds the κB element of the IL6 promoter: the substitution S425G reduces its occupancy, whereas a mutant with disturbed DNA recognition (K442A/R447A) is not recruited at all [44]. Repression of a reporter carrying a region of the IL6 promoter proceeds without a preceding activation of AP-1 and is not reproduced by the mineralocorticoid receptor [43].	Astrocytes are the main source of mediators of innate immunity in the CNS, including IL-1β, IL-6, and the chemokines CCL2, CXCL1, CXCL10, and CXCL12; activated microglia trigger a secondary cascade in which IL-1β induces TNF-α and IL-6 [241].	The mechanism is described in more detail than for the other genes of the table, but all the experiments were performed on cells that are not specific to the brain: RAW 264.7, MCF-7, and U-2 OS.
IL1B	Pro-inflammatory cytokine	The rate of activation of this gene is determined by the initiation of transcription: in primary macrophages there is no paused RNA polymerase II at the Il1b promoter, and dexamethasone suppresses its loading and prevents the LPS-induced acetylation of histone H3 in the area of the transcription start [242]. There is also a post-transcriptional level: in monocyte-like U937 cells, dexamethasone at 10 nM completely blocks the accumulation of IL-1β mRNA, whereas at 500 nM it suppresses the synthesis of IL-1 after stimulation without changing the level of mRNA, through a tran-	IL-1β is a product of activated microglia, secreted with the participation of the inflammasome, and one of the main mediators of astrocytes; it also triggers the production of TNF-α and IL-6 [241].	The gene belongs to the class with limiting initiation, and therefore the corepressor GRIP1, which is necessary for paused genes, is not involved here; see the rows for TNF and CCL2.

		sient increase of cAMP; the effect is mediated by GR and is abolished by progesterone [243].		
TNF (TNF-α)	Pro-inflammatory cytokine	The gene is regulated at the elongation step: a paused RNA polymerase II stands at its promoter, and GR together with the corepressor GRIP1 maintains the pause by accumulating the NELF factor; in macrophages lacking NELF the repression disappears [242]. In macrophages treated with LPS, the regulatory regions of Tnf bind GR, p65, and c-Jun simultaneously [41]. A separate post-transcriptional mechanism is the induction of tristetraprolin, which destabilizes TNF-α mRNA [62].	During inflammation, microglia produce large amounts of TNF-α; at high concentrations it disturbs the ability of astrocytes to remove glutamate, which leads to the death of neurons by the mechanism of excitotoxicity [241].	The TNF gene and the CCL2 gene are suppressed in one and the same way, by maintenance of the pause, and this distinguishes them from IL1B.
CCL2 (MCP-1)	Pro-inflammatory chemokine	In the CCL2 promoter there is a κB element with a hidden GR half-site; repression requires direct binding of GR to DNA, since the mutant K442A/R447A loses it, and GR recognizes this element as a monomer, without dimerization [44]. Like TNF, the gene is regulated at the elongation step: GR together with GRIP1 maintains the pause of RNA polymerase II [242]. In macrophages, Ccl2 is suppressed by dexamethasone, and its regulatory regions bind GR, p65, and c-Jun [41].	CCL2 is one of the chemokines released by astrocytes, the main source of mediators of innate immunity in the CNS [241].	Repression is carried out by monomeric GR: dimerization is not required for it, unlike activation through the classic GRE.
IL8 (CXCL8)	Pro-inflammatory chemokine	In the κB element of the IL8 promoter a hidden GR half-site has been found, the structure of the complex of the DNA-binding domain of GR with this element has been obtained, and repression requires direct binding to DNA [44]. At the same time, the substitution S425G, which reduces the occupancy of GR at the κB elements of IL6 and ICAM1, does not affect IL8, so that the effects of the mutations are gene-specific [44].	There are no direct data on the CNS in the works cited here. Rodents have no ortholog of IL8, and its functions are performed by CXCL1 and CXCL2; among the chemokines of astrocytes, CXCL1, CXCL10, and CXCL12 are named, but not CXCL8 [241].	The mechanism has been shown on human cells outside the nervous system. The row is not applicable to rodents, which have no ortholog of IL8.
RELB	Subunit of NF-κB (the alternative pathway)	The RELB promoter contains a κB element that is bound by the DNA-binding domain of GR;	There are no direct data on the CNS in the works cited here.	The only transcription factor in the table rather

		GR suppresses the activity of a reporter carrying this promoter, whereas a mutant with disturbed DNA recognition loses the effect [44].		than a secreted mediator: here GR suppresses not the inflammatory signal itself but a component of the alternative NF-κB pathway.
ICAM1	Adhesion molecule	In the κB elements of the ICAM1 promoter, hidden GR half-sites have been found; the occupancy of GR decreases upon the substitution S425G, and a mutant with disturbed DNA recognition loses the repression. In MCF-7 cells, GR and p65 compete for the ICAM1 promoter: dexamethasone reduces the recruitment of p65 [44].	There are no direct data on ICAM1 in the brain in the works cited here; it is known only that inflammatory cytokines reduce the barrier function of the blood-brain barrier and enhance the recruitment of leukocytes from the blood [241].	The only adhesion molecule in the table: through it GR influences not the production of mediators but the permeability of the vascular wall and the migration of leukocytes.
IL1R1 / IL1R2	IL-1 receptors	This is the only row in which GR does not suppress but enhances. In human glioblastoma U-87 MG cells there is a high-affinity IL-1 binding site (Kd about 104 pM, about 1200 sites per cell); incubation with cortisol or dexamethasone increases the expression of the receptor within as little as 6 h, and this effect is abolished by cycloheximide and by actinomycin D, that is, it requires transcription and translation; a GR antagonist was not used, and therefore the participation of the receptor itself in the induction has not been proved [244]. After preincubation with glucocorticoids, IL-1 induces the release of IL-6 more strongly, whence the conclusion of the authors: glucocorticoids may enhance and prolong the action of IL-1 in the CNS. On primary mouse astrocytes, dexamethasone increases the binding to IL-1RII [245], and in macrophages dexamethasone induces Il1r2, the decoy receptor [41]. An increase of the expression of cytokine receptors by	IL-1 receptors are expressed in neurons, astrocytes, microglia, and the endothelium of the brain and determine the sensitivity of brain cells to IL-1 [245].	What is shown here is the action of the hormone and not of the receptor: a GR antagonist was not used in the work of Gottschall et al. [244]. One should also distinguish IL-1RI from the decoy receptor IL-1RII, since an increase of each has the opposite biological meaning.

		glucocorticoids has been described as a general phenomenon [246].		
TSLP	Epithelial pro-inflammatory cytokine	In the TSLP promoter an inverted nGRE has been described that binds two GR monomers in the head-to-tail orientation; a complex with the corepressors SMRT and NCoR assembles on it and recruits the deacetylases HDAC2 and HDAC3. The binding requires an agonist and is prevented by RU486 [38]. The crystal structure of the DNA-binding domain of GR on this element showed that dimerization on it is impossible [48].	TSLP is a predominantly epithelial cytokine: skin, airways, intestine, thymus; there are no data on a significant expression in microglia and astrocytes [247].	The most thoroughly characterized nGRE, but the transfer of the mechanism to nervous tissue remains an assumption.
iNOS (NOS2)	Enzyme that produces NO	In macrophages treated with LPS, Nos2 is one of the genes suppressed by dexamethasone, and its regulatory regions bind GR together with p65 and c-Jun; at the same time, the direction of the effect cannot be predicted from the set of bound factors [41]. iNOS is also included in the list of genes whose repression through GR has been analyzed in the literature [62].	Glial cells produce nitric oxide along with derivatives of arachidonic acid and IL-6 [245].	The only enzyme in the table: GR suppresses not the mediator itself but its production, and therefore the effect covers the whole pool of nitric oxide. The mechanism has been established on macrophages, whereas in the brain the main source of NO during inflammation is the glial cells.

Note to the table. The assignment of a gene to one or another type of regulatory element does not predict the direction of the effect: in macrophages, GR both activates and represses genes at all types of region, and the outcome is determined by the context [41]. The role of the receptor itself has been proved by a receptor-level control, that is, by a GR mutant, by the occupancy of GR according to ChIP, or by an antagonist, in all the rows except IL1R1, where only inhibitors of transcription and translation were used. Almost all the mechanisms have been established on cells that are not specific to the brain, and therefore the column "Data on the CNS" shows for which genes the transfer to nervous tissue rests on data of its own and for which it remains an assumption.

Table S2. Anti-inflammatory factors: mechanisms of their regulation by the glucocorticoid receptor

Gene	Type	Mechanisms of regulation through GR	Data on the CNS	Comment
IL10	Anti-inflammatory cytokine	The induction does not proceed through the binding of GR to a GRE: the potential GRE motif is not required for the response, whereas mutation of the STAT motif at position −120 of the promoter sharply reduces transactivation, a dominant negative STAT3 blocks the induction, and chromatin immunoprecipitation shows the recruitment of STAT3 to the promoter. This has been shown on primary human CD19+ B lymphocytes and on a B cell line [151]. In human monocytes the participation of the receptor has been proved in a different way: dexamethasone at physiological doses increases the constitutive production of IL-10 at the level of mRNA and of protein, the effect is dose-dependent and proportional to the activity of the steroid and is completely abolished by the antagonist RU486; in T and B lymphocytes, in mature macrophages, and in dendritic cells there is no such effect [248].	IL-10 is produced by microglia upon activation through TLR2, TLR4, and TLR9 [249]. In microglia stimulated with LPS, IL-10 suppresses the production of IL-12, TNF-α, and nitric oxide [250]. The loss of IL-10 shifts microglia towards a pro-inflammatory phenotype [251], and in models of amyotrophic lateral sclerosis the level of microglial IL-10 determines the time of disease onset [252].	The mechanism depends on the cell type. In B lymphocytes it proceeds without the participation of a GRE, through STAT3, and the participation of the receptor itself was not tested there either with an antagonist or with a knockdown [151]. In monocytes the dependence on the receptor has been proved with an antagonist [248]; however, direct binding of GR to the IL10 promoter has not been shown in any of the works.
IL1RN (IL-1Ra)	Antagonist of the IL-1 receptor	The direction of the effect depends on the cell type. In monocytes stimulated with endotoxin, activation of GR dose-dependently suppresses the mRNA and the secretion of IL-1Ra: the effect is abolished by the GR antagonist RU 38486 [153]. In a human airway epithelial cell line, corticosteroids, on the contrary, induce the intracellular type I isoform of IL-1Ra [152]. There is no mapping of a GRE in the regulatory region of IL1RN.	There are no direct data on the CNS in the works cited here.	The direction of the effect is opposite in different cells. In monocytes the role of the receptor has been proved with an antagonist, and Arzt et al. [153] explicitly exclude the induction of IL-1Ra from the anti-inflammatory mechanism of glucocorticoids; for the epithelium there is no receptor-level control.
ANXA1 (annexin A1)	Protein effector of the anti-inflammatory action	The role of the receptor has been proved for the rapid, non-genomic step: in a line of human folliculostellate pituitary cells,	In rat C6 astrocytoma cells, dexamethasone increases the	The receptor acts on ANXA1 at two levels at once: the rapid non-genomic one,

		dexamethasone within 30 min causes serine phosphorylation of ANXA1 and the transfer of the phosphorylated protein to the cell surface, and this is blocked by the GR antagonist mifepristone but not by actinomycin D or cycloheximide; PKC, PI3K, and MAPK participate in the transfer. The slow genomic response, that is, an increase of ANXA1 mRNA and protein, develops later, from 2 h [253]. The protein then suppresses phospholipase A2 and the migration of leukocytes; this is historically the first described mechanism of the anti-inflammatory action of glucocorticoids [144,148]. The activation of the ANXA1 promoter under the action of dexamethasone, mediated by GR, has been shown in T lymphocytes as well: it is disturbed upon a deficiency or an inactivation of the kinases Zap70 and Lck [149]. Direct mapping of a GRE in the ANXA1 promoter and ChIP data for GR are nevertheless absent.	level of the mRNA and of the intracellular protein of lipocortin 1 and increases its secretion without signs of cytotoxicity; there is no receptor-level control in that work [150].	that is, phosphorylation of the protein and its transfer to the membrane, and the slow genomic one. Functional support is provided by the knockout of annexin itself: the inflammatory response is enhanced and the sensitivity to glucocorticoids is reduced [107,108].
DUSP1 (MKP-1)	MAPK phosphatase, a suppressor of p38 and JNK	In A549 cells, GR regulates DUSP1 at the step of transcription initiation; chromatin immunoprecipitation localizes the binding of GR between −1421 and −1118 from the transcription start site, where a consensus GR binding site is located within an organized nucleosomal region. A second GRE is located at about −4.6 kb, and mutation of either of the two halves the hormonal response [160]. The maximal induction is achieved upon simultaneous activation of GR and of inflammatory signaling [254]. The protein dephosphorylates p38 and JNK and, in addition, enhances the destabilizing activity of tristetraprolin towards the mRNAs of Tnf, Il1b, and others [144].	The data are contradictory. Overexpression of MKP-1 protects neurons from death upon hypoxia and reoxygenation by limiting JNK signaling [161]. On the contrary, in prenatal stress the complex of GR with the acetyltransferase P300 increases MKP1 in the hippocampus of the offspring, suppressing the ERK/CREB/TrkB pathway; this re-	The role of the receptor has been proved from two sides: by the occupancy of GR according to ChIP and by mutagenesis of the GRE in a cell model, and by mifepristone in the brain. At the same time, the direction of the effect in the brain depends on the model, and it cannot be considered protective.

			duces neurogenesis and leads to depression-like behavior, and mifepristone abolishes the effect [163].	
TNFAIP3 (A20)	Ubiquitin-modifying enzyme, a negative regulator of NF-κB	The regulation proceeds not through the promoter but through an intronic enhancer, where GR and NF-κB cooperate. In airway epithelial cells such cooperation covers a whole group of genes that limit inflammation: TNFAIP3, TNIP1, ZC3H12A, IRAK3, BIRC3, SPHK1, and SERPINA1 [254].	There are no direct data on the CNS in the works cited here.	The induction requires not only the activation of GR but also the inflammatory signal itself: without NF-κB the cooperation at the intronic enhancer does not come about.
ZFP36 (TTP)	RNA-binding protein that accelerates the degradation of cytokine mRNAs	The direction of the effect is opposite in different cells, and both sides have been proved with a GR antagonist. In A549 lung epithelial cells and in rat tissues, activation of GR increases the level of the mRNA and of the protein of tristetraprolin; the induction is abolished by RU486 and is blocked by actinomycin D but not by cycloheximide, and run-on experiments confirm a transcriptional mechanism. The suppression of TNF-α mRNA by the glucocorticoid disappears upon knockdown of tristetraprolin, that is, the protein is necessary for this effect [255]. In LPS-activated macrophages, on the contrary, dexamethasone and the dissociated steroid RU24858 suppress the induced expression of tristetraprolin; the suppression is abolished by mifepristone, does not depend on a GRE, and apparently proceeds through histone deacetylation [256]. The protein binds AU-rich elements in the 3′ untranslated regions of the mRNAs of pro-inflammatory cytokines, and its destabilizing activity is enhanced by the phosphatase DUSP1 [144].	There are no direct data on the CNS in the works cited here.	The sign of the effect depends on the cell type and on whether the cell is activated, and a GRE is not involved in either of the works. This is a clear example of the general rule: the outcome is determined not by the element but by the context.

TSC22D3 (GILZ)	Anti-inflammatory protein, a mediator of the effects of GR	The gene is activated by GR through a canonical GRE: chromatin immunoprecipitation shows the occupancy of GR at this element along with FKBP5 and SGK1 [43,44]. The same element is used by both MR and GR [36]. Unlike TNFAIP3, cooperation with NF-κB is not required for the induction of TSC22D3 [254]. The protein binds c-Fos and c-Jun, preventing AP-1 from landing on DNA [155], and suppresses NF-κB [62]; the anti-inflammatory effect has been shown on T lymphocytes, on dendritic cells, and in endotoxemia [154].	A decrease of GILZ expression is associated with an increased risk of PTSD symptoms in male mice and in men [157]. There are no data on GILZ in particular types of brain cells in the works cited here.	One of the few genes of the table with a proved canonical GRE and a direct occupancy of GR. The anti-inflammatory action of the protein itself has been shown on peripheral cells; there are no data on GILZ in particular types of brain cells.
IRAK3 (IRAK-M)	Negative regulator of TLR and IL-1R signaling	Activation of GR and a bacterial stimulus increase IRAK-M synergistically: p65 and GR mutually enhance each other's binding to the IRAK-M promoter. The binding of dimerized GR to a GRE is necessary, since the dissociated ligand CpdA does not produce induction although it does suppress NF-κB. In mice lacking IRAK-M, the glucocorticoid does not improve survival upon infection [257]. The same cooperation of GR and NF-κB at enhancers has been described as a general mechanism [254]. The protein blocks the signal from TLR and IL-1R by preventing IRAK1/4 from separating from the MyD88 complex [258].	There are no direct data on the CNS in the works cited here.	The induction requires dimerization of GR: a dissociated ligand that retains the suppression of NF-κB does not induce IRAK-M. This separates two actions of the receptor, the suppression of NF-κB and the activation of the gene.

Note to the table. The role of the receptor itself has been proved by a receptor-level control, that is, by an antagonist, by a mutant, by a knockout, by the occupancy of GR according to ChIP, or by mutagenesis of a GRE, for IL10 and IL1RN in monocytes, for ANXA1, DUSP1, TNFAIP3, ZFP36, TSC22D3, and IRAK3. Only the action of the hormone has been shown for IL1RN in the epithelium. Direct binding of GR to the regulatory region of the gene has not been shown for IL10, ANXA1, and ZFP36. For five genes out of eight, no direct data on the CNS were found in the available literature, and this is indicated in the corresponding cells. Finally, for TNFAIP3, IRAK3, and in part DUSP1, activation requires not only the ligand but also the inflammatory signal itself: GR and NF-κB cooperate at enhancers [254].
